# Low-Grade Fibromyxoid Sarcoma and Related Subtypes: A Systematic Review and Pooled Analysis of 773 Cases

**DOI:** 10.3390/cancers18030364

**Published:** 2026-01-23

**Authors:** Gitte G. J. Krebbekx, Elisabeth A. Kleine, C. Dilara Savci-Heijink, Diederik T. Meijer, Donner, Robert Hemke, Floortje G. M. Verspoor

**Affiliations:** 1 Department of Orthopedic Surgery and Sports Medicine, Amsterdam Movement Sciences, Amsterdam UMC, University of Amsterdam, Meibergdreef 9, 1105 AZ Amsterdam, The Netherlands; 2 Amsterdam Movement Sciences, Rehabilitation & Development, Meibergdreef 9, 1105 AZ Amsterdam, The Netherlands; 3 Cancer Center Amsterdam, Meibergdreef 9, 1105 AZ Amsterdam, The Netherlands; 4 Department of Pathology, Amsterdam UMC, University of Amsterdam, Meibergdreef 9, 1105 AZ Amsterdam, The Netherlands; 5 Amsterdam Institute for Immunology and Infectious Diseases, Cancer Immunology, Meibergdreef 9, 1105 AZ Amsterdam, The Netherlands; 6 Department of Radiology and Nuclear Medicine, Amsterdam Movement Sciences, Amsterdam UMC, University of Amsterdam, Meibergdreef 9, 1105 AZ Amsterdam, The Netherlands

**Keywords:** low-grade fibromyxoid sarcoma, soft tissue sarcoma, MUC4, recurrence, systematic review

## Abstract

Background: Low-grade fibromyxoid sarcoma (LGFMS) is a rare malignant fibroblastic tumor that often appears deceptively benign. Accurate diagnosis is challenging due to its variable morphology and low mitotic activity. This systematic review provides a comprehensive overview of LGFMS and its subtypes. Methods: A systematic search of PubMed and Embase up to September 2025 identified 273 studies, complemented by four institutional cases from Amsterdam UMC. Individual patient data were pooled to analyze clinical presentation, diagnostic approaches, treatment modalities, and outcomes. Results: In total, 773 patients were included, with a median age of 35 years and equal gender distribution. Tumors were predominantly deep-seated (80%), most commonly located in the thigh or pelvis. MUC4 positivity (96%) and FUS-CREB3L2 fusion (47%) were the most consistent diagnostic markers. Surgery was the mainstay of treatment (98%), with R0 resection achieved in 36% of cases and R1 in 15%. Adjuvant therapies, including chemotherapy and radiotherapy, were rarely used and showed limited efficacy. After a median follow-up of 3.0 years, 19% developed local recurrence and 21% developed metastases. R0 resections were associated with significantly better recurrence-free survival than R1 resection (*p* < 0.05). Conclusions: LGFMS exhibits indolent histology but potential for late recurrence and metastasis, warranting prolonged radiological follow-up and multicenter studies to evaluate adjuvant strategies.

## Simple Summary

Low-grade fibromyxoid sarcoma (LGFMS) is a rare soft-tissue sarcoma that often appears harmless under the microscope, yet can recur or metastasize many years after surgery. Because of its subtle histological appearance, diagnosis can be difficult and is often confirmed by the presence of MUC4 protein and FUS-CREB3L2 gene fusion. We systematically reviewed all published cases of LGFMS and included four additional patients treated at our institution. Among 773 patients, wide (R0) surgical resection offered the best local control, while chemotherapy and radiotherapy provided little benefit. Nearly one in five patients developed local recurrence or metastasis, sometimes after a long disease-free interval. These findings highlight the need for careful diagnosis, complete resection, and long-term radiological follow-up in all patients with LGFMS.

## 1. Introduction

Low-grade fibromyxoid sarcomas (LGFMSs) are rare, slow-growing, and typically painless malignant tumors that arise in the deep soft tissues, accounting for approximately 0.6% of all soft tissue sarcomas [1]. It occurs predominantly in adults between 20 and 50 years of age, with an estimated incidence of 0.2 per million individuals [2]. According to the World Health Organization (WHO, 2020) classification, LGFMS belongs to the group of malignant fibroblastic tumors [3]. The entity was first described by Evans in 1987, who reported two adolescent cases with deceptively benign-appearing lesions, low cellularity, minimal mitotic activity, yet pulmonary metastases [4]. Histologically, LGFMS consists of bland-appearing spindle cells arranged in whorled or swirling fascicles, often displaying alternating fibrous and myxoid areas. In 1997, Lane et al. introduced the hyalinizing spindle cell tumor with giant rosettes (HSCTGR), which shares similar morphology and clinical behavior [5]. The sclerosing epithelioid fibrosarcoma (SEF), described by Meis-Kindblom in 1995, represents a more aggressive variant with overlapping features, and since 2011, hybrid tumors combining LGFMS, HSCTGR, and SEF patterns have also been recognized [6].

Cytogenetic studies have become essential for distinguishing fibroblastic neoplasms [7]. The most frequent fusion genes involve FUS and EWSR1, both of which encode RNA-binding proteins, and CREB3L1/2, which encode DNA-binding proteins [8,9]. The FUS-CREB3L2 t(7;16) fusion is characteristic and occurs in the vast majority of cases [10], whereas FUS-CREB3L1 t(11;16) and EWSR1-CREB3L1 t(11;22) are less common variants [11]. These translocations generate chimeric transcription factors that are believed to drive tumorigenesis.

Histopathological diagnosis was historically limited to non-specific mesenchymal markers such as vimentin [1]. Since 2012, MUC4 has been identified as a highly sensitive and specific immunohistochemical marker, expressed in nearly all LGFMS [11,12]. MUC4 is a transmembrane glycoprotein involved in cell-signaling and differentiation, normally expressed in endodermal tissue [13]. Misdiagnosis due to sampling errors or limited biopsies may lead to inadvertent, incomplete resections, thereby increasing the risk of local recurrence and requiring re-excision, often with significant functional morbidity [1,14].

Magnetic resonance imaging (MRI) typically demonstrates a heterogeneous lesion with fibrous component showing low signal intensity on both T1- and T2-weighted sequences, and a myxoid component showing high T2 signal intensity and a gyriform pattern or laminated enhancement pattern after gadolinium administration [15,16].

The mainstay of treatment is wide surgical resection, given the risk of both local recurrence and distant metastasis [1]. Adjuvant radiotherapy, chemotherapy, and immunotherapy have shown minimal or no impact on tumor control or survival [2,17,18,19]. Long-term follow-up is therefore essential, although reported data remain limited, with only a minority of patients monitored beyond three years [1]. Despite its deceptively benign histologic appearance, LGFMS carries a significant potential for late recurrence and metastasis, making accurate diagnosis and adequate surgical management critical [1]. Current evidence is derived mainly from numerous case reports and small retrospective series, resulting in fragmented knowledge and a lack of standardized treatment recommendations.

A comprehensive synthesis of the available literature is thus required to better define the clinical spectrum, improve diagnostic accuracy, and inform evidence-based management. To address this gap, we conducted a systematic review and included a consecutive case series from our own institution. The primary objective was to evaluate current evidence regarding the clinical presentation, diagnostic features, treatment, and outcomes of LGFMS and its related subtypes. As a secondary objective, we compared outcomes between R0 and R1 resections to assess the prognostic relevance of surgical margin status.

## 2. Method

### 2.1. Study Design and Overview

This study comprised a systematic literature review including institutional cases. All methods followed the STROBE [20] and PRISMA [21] guidelines, with study quality assessed using the Joanna Briggs Institute (JBI) Checklist for Case Series [22].

The study was registered at Amsterdam UMC (ID: 120491) and PROSPERO (ID: CRD42022310606). The protocol was developed a priori to ensure methodological transparency and reproducibility. Ethical approval for the institutional component was covered under an existing Medical Ethics Review Committee registration (No. 2025.0109).

### 2.2. Search Strategy and Study Selection

A systematic search of PubMed/MEDLINE and Embase was performed up to September 2025 using the following search terms:

“fibromyxoid sarcoma” OR “LGFMS” OR “Evans tumor” OR “hyalinizing spindle cell tumor with giant rosettes” OR “HSCTGR”.

This search yielded 607 unique records.

Studies were eligible if they reported on LGFMS, HSCTGR, or SEF hybrid tumors and were published in English, French, German, Italian, or Spanish, without date restrictions. Exclusion criteria included review articles, series lacking individual patient data, and studies describing only diagnostic or therapeutic techniques without outcome data.

Two reviewers (GGJK and EAK) independently screened all titles, abstracts, and full texts using Rayyan (Qatar Computing Research Institute, Doha, Qatar) [23]. Discrepancies were resolved by consensus. Reasons for exclusion were documented at each stage.

### 2.3. Institutional Cases

In addition to the literature review, four consecutive patients with histologically confirmed LGFMS treated at Amsterdam UMC between 2018 and 2025 were included. Clinical, radiological, and histopathological data were extracted retrospectively from the institutional database. All patients provided informed consent for data use and publication. Institutional cases were identified from the Musculoskeletal Tumor (MUST) registry and are described in summary here; detailed case descriptions are provided in Supplementary Materials S1.

To preserve methodological consistency, these cases were analyzed together with the literature-derived cohort as part of the pooled dataset.

### 2.4. Quality Assessment

The methodological quality of all cohorts with ≥5 cases was independently assessed by two reviewers using the JBI Critical Appraisal Checklists for case series and observational studies [24,25]. Smaller case reports were not formally scored due to the inherent risk of bias and design heterogeneity. Each JBI was rated as “yes” (low risk of bias), “no” (high risk of bias), or “unclear/not applicable”. No automation or IA-based tools were used in this process. Any disagreement between reviewers was resolved by consensus discussion.

### 2.5. Data Extraction and Synthesis

The primary objective of this study was to evaluate the clinical presentation, diagnostic characteristics, treatment strategies, outcomes, and follow-up of LGFMS and related subtypes. The secondary objective was to compare recurrence-free survival (RFS) between patients who underwent R0 and R1 resections.

Data were manually extracted into a structured database. Extracted variables included: demographics (age, sex), tumor features (anatomical site, depth, size, symptoms, imaging characteristics), histopathology and immunohistochemistry features (MUC4 expression, cellularity, mitotic index), cytogenetics (FUS-CREB3L2, FUS-CREB3L1, and EWSR1-CREB3L1 fusions), treatment (initial diagnosis, inadvertent resections, resection margins, adjuvant therapy), outcomes (postoperative complications, recurrence, metastasis, and follow-up duration.

Tumor size was primarily extracted from pathology reports; if unavailable, measurements from imaging were used. Resection margins were standardized according to the AJCC 7th edition (R0–R2) [26]. Diagnoses were categorized as LGFMS, HSCTGR, SEF hybrid, or unclassified fibromyxoid sarcoma (UFMS). When relevant data were missing or aggregated, corresponding authors were contacted to obtain individual-level information.

### 2.6. Statistical Analysis

All statistical analyses were conducted using IBM SPSS Statistics, version 26 (IBM Corp., Armonk, NY, USA). Categorical variables (e.g., resection margin, histopathological subtype) were analyzed using Pearson’s Chi-square test, following verification of test assumptions. Continuous variables (e.g., age, tumor size) were compared using the Mann–Whitney U test or independent-samples *t*-test, depending on data distribution. Descriptive statistics (frequencies, medians, and interquartile ranges) summarized demographic, pathological, and treatment characteristics.

Recurrence-free survival (RFS) was analyzed using Kaplan–Meier survival curves, comparing R0 and R1 resections; statistical significance was defined as *p* < 0.05. Survival plots were generated in MATLAB R2023b (MathWorks, Natick, MA, USA).

## 3. Results

### 3.1. Study Selection and Characteristics

**Figure 1:**
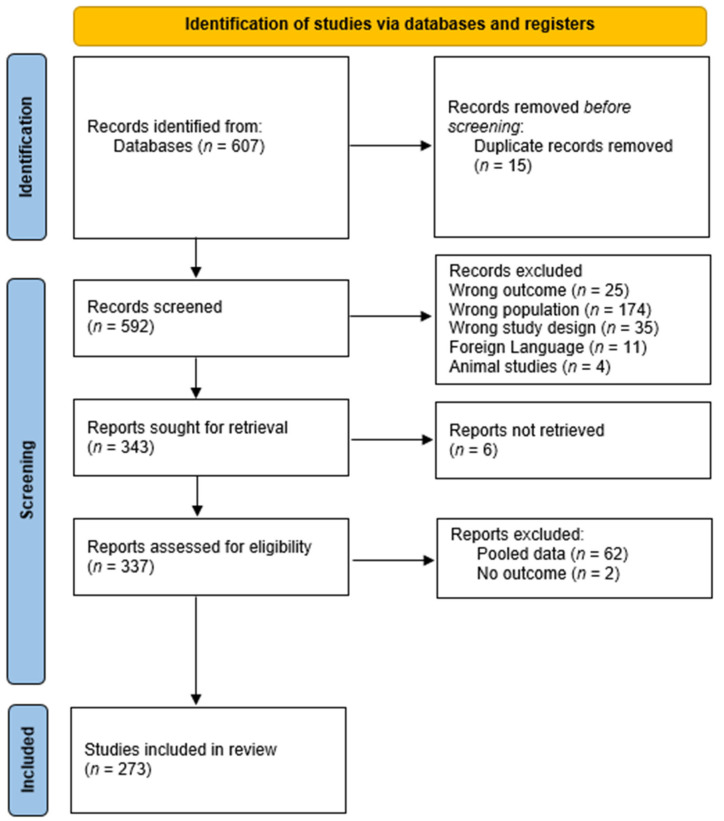
PRISMA flow diagram for inclusion.

The systematic search identified 607 unique studies, of which 273 met the inclusion criteria and were included in the pooled analysis (Supplementary Materials S2) After screening and full-text review, 333 articles were excluded due to duplication, foreign language, wrong outcome, wrong population or study design, lack of individual patient data, or insufficient outcome information (Figure 1). In total, 773 individual patients were analyzed, including four additional institutional cases from Amsterdam UMC (Supplementary Materials S1). Most included studies were retrospective single-center case series or isolated case reports (244 small case series (*n* < 5) and 30 larger case series), with publication years ranging from 1987 to 2025. The median follow-up across all series was 3.0 years (IQR 1.0–6.1) (Table 1).

### 3.2. Critical Appraisal

Across all 11 JBI domains, an average of 88% of checklist items were rated as low risk of bias (“yes”), 9% as unclear (“?”), and 3% as high risk of bias (“no” = red). Nearly all studies (97%) clearly defined inclusion criteria, exposure variables, and outcome measures. Study populations were generally representative, with most cohorts derived from institutional or national sarcoma registries and based on pathologically confirmed diagnoses of LGFMS or related subtypes. Diagnostic confirmation using MUC4 immunopositivity and/or FUS-CREB3L2 fusion was reported in 93% of studies.

Outcomes were assessed using valid and reproducible criteria in approximately 90% of studies. Potential confounders were identified and addressed in 75%, while 25% provided insufficient information or lacked statistical adjustment. Adequate follow-up to detect recurrence or metastasis was reported in 85% of studies, and loss to follow-up was minimal or appropriately accounted for in 83%. Only three studies (7%) demonstrated incomplete follow-up reporting.

Overall, 26 of 30 cohorts (87%) fulfilled at least nine of eleven JBI criteria, indicating high methodological robustness across the included evidence base. Minor limitations mainly related to incomplete control for confounding or lack of detailed statistical analysis due to small sample sizes. Importantly, no studies were excluded based on the JBI assessment, as all met acceptable quality standards for pooled synthesis. Detailed JBI domain scoring is provided in Supplementary Materials S3.

### 3.3. Patient Demographics and Tumor Distribution

**Figure 2:**
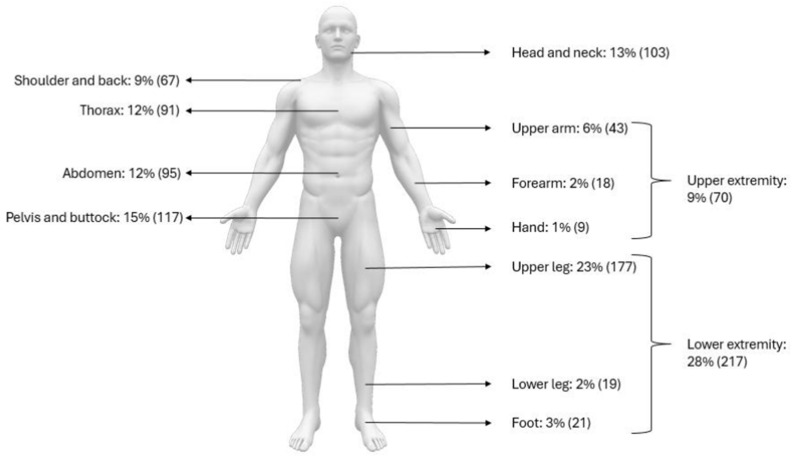
Anatomical distribution of LGFMS.

The median age at diagnosis was 35 years (IQR 21–49, range: neonatal [27] to 97 years [28]), with an equal sex distribution (50% females, *n* = 389). Tumors were predominantly deep-seated (80%, *n* = 475), and located in the lower extremity (43%, *n* = 334), most frequently in the thigh (23%, *n* = 177), followed by the upper extremity (18%, *n* = 138), pelvis/sacrum/buttock (15%, *n* = 117), thorax (12%, *n* = 91), and abdomen (12%, *n* = 95). Superficial lesions were uncommon (8%) and mainly affected the chest wall and abdominal wall. Reported tumor size (70%, *n* = 547) in maximum diameter was median 6.1 cm (IQR 3.8–10.0, range 0.7 cm–50 cm [28,29]). Detailed information and patient demographics and tumor distribution Table 1 and Figure 2.

### 3.4. Histopathology, Immunohistochemistry, and Cytogenetics

Histopathological evaluation in the included studies was performed on both fresh-frozen tissue and formalin-fixed, paraffin-embedded (FFPE) samples, frequently obtained from external institutions. Tumors typically demonstrated the classic biphasic architecture, composed of bland spindle cells arranged in whorled or swirling fascicles within alternating fibrous and myxoid zones, often with low cellularity, minimal atypia, and absent necrosis.

Immunohistochemical data were available for 428 patients (62%). The non-specific mesenchymal marker vimentin was positive in 198 cases (46%) and negative in one case [16] (0.5%), confirming its limited diagnostic specificity. MUC4, the key diagnostic marker for LGFMS, was analyzed in 428 cases and showed strong cytoplasmic positivity in 243 (57%), while only eight cases (3%) were negative. Additional immunostains, including β-catenin, CD34, calponin, caldesmon, desmin, EMA, ERG, keratin, p53, p63, S100, α-SMA, and SOX10, were inconsistently reported and largely negative across series. Ki-67 nuclear labeling indices were generally low (<5%), consistent with the indolent proliferative activity of LGFMS.

Cytogenetic testing was performed in 318 patients (41%), of whom 268 (84%) demonstrated a detectable gene rearrangement. The FUS gene was frequently altered, showing rearrangement or translocation in 263 cases (83%). The predominant fusion was FUS-CREB3L2 (47%, *n* = 148). A smaller subset showed EWSR1 rearrangements (8%, *n* = 24), with only one case confirming an EWSR1–CREB3L2 translocation [30]. In 31 cases, the FUS gene showed no rearrangement, and no other alternative fusions were analyzed.

Molecular diagnostics were primarily conducted by fluorescence in situ hybridization (FISH) to detect FUS gene rearrangements, while selected series used reverse transcriptase polymerase chain reaction (RT-PCR) or targeted next-generation sequencing (NGS). The Archer FusionPlex Sarcoma Panel was applied in several contemporary studies to identify FUS–CREB3L2 fusion transcripts among a panel of soft-tissue tumor–associated genes [31].

### 3.5. Clinical Presentation and Diagnostic Accuracy

Clinical symptoms were reported for 257 patients (33%). The majority presented with a slow-growing mass (27%, *n* = 69) or, less frequently, a rapidly enlarging lesion (9%, *n* = 24). Pain was absent in most cases (29%, *n* = 75), while 19% (*n* = 48) reported localized pain or discomfort. Median symptom duration before diagnosis was 1 year (IQR 0.5–4.0) (Table 1), although the longest reported symptom duration reached 34 years [32].

An initial diagnosis was documented in 203 patients (26%). Correct identification by imaging or biopsy was achieved in 54% of cases as LGFMS (*n* = 110) and in 38% as low-grade sarcomas (*n* = 77); the remaining cases were frequently misdiagnosed as benign lesions (47%, *n* = 95).

Misclassification occurred after biopsy (*n* = 37, including 26 FNA), imaging alone (*n* = 14), clinical assessment (*n* = 11), or frozen-section analysis (*n* = 2).

A total of 101 patients (32%) underwent an unplanned (“WHOOPS”) resection, defined as inadvertent excision without prior diagnosis or oncological planning. Of the 29 cases in which re-excision was reported, 25 (86%) achieved secondary R0 margins upon definitive surgery.

Following resection and histopathological review, 602 tumors (78%) were confirmed as LGFMS, 96 (12%) as HSCTGR, 44 (6%) as LGFMS/SEF hybrid, 10 (1%) as HSCTGR/SEF hybrid, 4 (<1%) as pure SEF variants, and 18 (2%) as unclassified fibromyxoid sarcomas (UFMSs).

### 3.6. Radiological Features

Imaging findings were reported for 273 patients, of whom 25 (14%) received a diagnosis based on imaging alone. Among these, 13 cases (52%) were misclassified as benign. The imaging modalities used included CT in 127 patients (47%), MRI in 109 (40%), ultrasound in 44 (16%), and FDG–PET/CT in 15 (6%).

Lesions typically appeared as well-defined, heterogeneous soft-tissue masses hypo- to isodense on CT, hypoechogenic on ultrasound. On MRI, tumors demonstrated mixed fibrous and myxoid signal patterns, with low T1 and high T2 signal intensity, and heterogeneous post-contrast enhancement. Occasional calcifications, hemorrhagic areas, or adjacent bone involvement were described. FDG–PET/CT findings were variable, showing low to moderate metabolic activity consistent with the tumor’s low-grade biology, although rare hypermetabolic cases were reported.

### 3.7. Treatment and Adjuvant Therapy

Surgical excision was the primary treatment modality in 98% (*n* = 760) of patients, 2% (*n* = 13) had unresectable or metastatic disease [2,30,31,33,34]. Resection margins were reported as R0 in 36% (*n* = 280), R1 in 15% (*n* = 113), R2 in 3% (*n* = 19), and unknown in 47% (*n* = 362) (Table 1). Intraoperative or postoperative complications were reported in 25 patients (3%). The severity of postoperative events varied from minor wound infections [35] to major systemic complications such as pulmonary embolism [36]. Some patients required emergency reoperations for procedure-related obstructions or vascular events, including bowel resection [37], ureteric stenting [38], nephrectomy [39], and above-knee amputation following thrombosis of the external iliac artery [40].

Adjuvant therapy was administered in 17% of patients (*n* = 128), predominantly radiotherapy 10% (*n* = 79) and chemotherapy (6%, *n* = 49). Reported agents included cyclophosphamide plus fecitabine [18], docetaxel [41], adriamycin [41], trabectedin [2], pazopanib [41,42], sunitinib [19] and anastrozole [41]. One patient experienced a coma secondary to Sunitinib therapy, which resolved after discontinuation of the drug [21].

### 3.8. Oncological Outcomes and Follow-Up

Follow-up status was available for 71% of patients (*n* = 545). At last follow-up, 422 (55%) had no evidence of disease (NED), 79 (10%) were alive with disease (AWD), 41 (5%) had died of disease (DOD), and 4 (0.5%) had died of other causes (DOOD) [43,44,45].

Thirty patients (4%) were lost to follow-up, while 198 (26%) had no follow-up information reported.

The overall median follow-up was 3.0 years (IQR 1.0–6.1). During follow-up, local recurrence occurred in 143 patients (19%), lung metastases in 62 (11%), and other distant metastases in 54 (10%). The median time to first recurrence was 3.1 (IQR 1.3–7) years, with the latest recurrence reported after 30 years [46]. The median time to local recurrence was 2.8 years (IQR 0.8–6.0), to lung metastasis 4.0 years (IQR 0.0–11.3), and to other metastases 2.3 years (IQR 0.0–9.3). Most patients (15%, *n* = 83) experienced a single recurrence, whereas 51 (9%) patients developed multiple recurrences, and 9 patients (2%) had ≥5 recurrences. Remarkedly, one patient had 17 recurrences [45] (Table 1). Local control and survival were not consistently improved by adjuvant therapy: no evidence of disease was observed in 5 patients (10%) treated with chemotherapy and in 21 (27%) of those treated with radiotherapy, compared with 442 patients overall (55%). Death of disease occurred in 4 patients (8%) after chemotherapy and in 5 (6%) after radiotherapy, compared with 41 patients (5%) overall. Local control and survival were better in superficial compared with deep tumors. No evidence of disease was observed in 258 patients (54%) with deep tumors and in 82 (67%) with superficial tumors, compared with 422 patients overall (55%). Death of disease occurred in 33 patients (7%) with a deep tumor and in 2 (2%) with a superficial tumor, compared with 41 patients (5%) overall.

The four institutional patients underwent wide or marginal resection without adjuvant treatment and remained free of disease recurrence or metastasis after a median follow-up of 3.1 years (range 1.1–5.1).

No significant differences were found between pathology-proven and unproven cases in survival, local recurrence, lung metastasis and distant metastases outcomes, allowing combined analysis of all R0 and R1 resection margin groups (Supplementary Materials S4).

The median follow-up time for R0 resections was 2.1 years (IQR 1.3–5.2) and for R1 resections was 2.9 (IQR 1.0–7.5) years. Overall survival was higher after R0 resection (96%, *n* = 226) than after R1 resection (89%, *n* = 69; *p* = 0.02). Local recurrence occurred less frequently following R0 resection (9%, *n* = 24) than R1 resection (24%, *n* = 27; *p* < 0.001). No significant differences were observed in the incidence of lung metastases (4% vs. 6%; *p* = 0.42) or other distant metastases (5% vs. 6%; *p* = 0.81). (Table 2)

**Figure 3:**
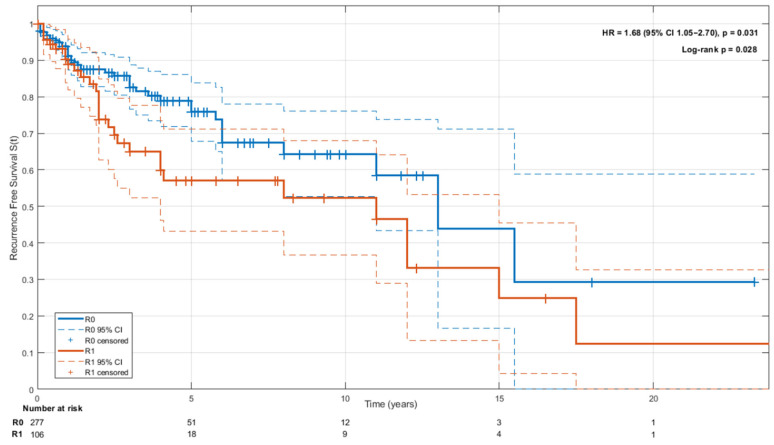
Kaplan–Meier analysis of recurrence-free survival (RFS) by resection margin status.

The Kaplan–Meier survival analysis (Figure 3) demonstrated significantly improved recurrence-free survival R0 compared to R1 resections. Median RFS was longer in the R0 group, with a hazard ratio (HR) of 1.68 (95% CI 11.05–2.70, *p* = 0.03). The log-rank test confirmed a significant difference between the two groups (*p* = 0.03). The 5-, 10-, and 15-year recurrence-free survival (RFS) rates were 57% (95% CI, 43–67%), 52% (36–68%), and 25% (4–49%) for patients with R1 resections, and 78% (71–83%), 64% (53–76%), and 29% (0–59%) for those with R0 resections, respectively.

Follow-up recommendations varied substantially between studies. Suggested surveillance modalities included whole-body imaging [27], MRI [47,48,49], CT [50,51,52,53,54], chest imaging [55,56,57,58,59], or combinations of local and distant imaging techniques [40,60,61]. The recommended surveillance duration ranged from several decades [32,55] to lifelong follow-up [59,62]. Proposed imaging intervals varied from every 3 months to every 6 months or annually

## 4. Discussion

Low-grade fibromyxoid sarcomas (LGFMSs) and their related subtypes occur across various locations, predominantly in young adults. These deep-seated soft tissue tumors usually present as painless, slowly enlarging lesions, often reaching considerable size before detection. Approximately 90% of tumors were deep-seated, a proportion slightly higher than previously reported (83%). Superficial tumors appear to have a more favorable prognosis, likely due to earlier recognition, smaller size, and facilitated excision, as demonstrated by Billing et al. [63]. Histopathological confirmation is based on morphology in combination with MUC4 immunopositivity and characteristic FUS–CREB3L2 or, less frequently, EWSR1-CREB3L1 translocations.

### 4.1. Diagnostic Challenges and Biopsy Technique

Diagnostic delay is common, resulting from the tumor’s indolent course and deceptively benign morphology. Fine-needle aspiration (FNA) frequently produced non-diagnostic or misleading results, leading to inadvertent resections and repeat surgery. Core-needle or open biopsy provides a higher diagnostic yield and should be preferred to ensure accurate molecular and immunohistochemical analysis before resection. The most frequent false-negative benign diagnoses included desmoid-type fibromatosis, nodular fasciitis, and peripheral nerve-sheath tumors, while false-negative malignant diagnoses often included solitary fibrous tumor and malignant fibrous histiocytoma [28,64,65,66].

A wide spectrum of imaging modalities was used for diagnosis and follow-up, including ultrasound, radiography, CT, PET-CT, and MRI. Although MRI and CT best delineate fibromyxoid tumors [15], imaging findings remain non-specific, and diagnosis should never rely solely on radiologic assessment. The combination of tissue biopsy with molecular testing remains the diagnostic gold standard.

### 4.2. Histopathology and Cytogenetics

Consistent with prior literature [67], our review confirmed MUC4 positivity in 96% of tested cases and FUS–CREB3L2 rearrangement in 47%. These rates are slightly higher than earlier series, reflecting improvements in molecular testing over time. The cytogenetic profile in our cohort—with 90% FUS and 6% EWSR1 rearrangements—was slightly higher than reported in previous studies. The French Sarcoma Group [10] identified FUS–CREB translocations in 81% of 59 patients, including several non-LGFMS sarcomas (four pure SEF cases), while the smaller cohort of Matsuyama et al. [68] (*n* = 16) demonstrated 84% positivity for FUS–CREB3L2.

EWSR1 rearrangements were rare, typically confined to pure SEF (90%) [11] and, in a few additional cases, observed in LGFMS/SEF hybrid tumors [27,35,36,69,70] and LGFMS/SEF hybrid tumors [33,34,37,38,39]. Beyond these canonical fusions, several novel rearrangements have been described (e.g., KMT2A–YAP1, HEY1–NCOA2, YAP1–TFE3), though their biological relevance remains uncertain. Together, these data emphasize that LGFMS represents a molecular and morphological spectrum, rather than a single uniform entity.

Several non-specific immunohistochemical markers, including α-SMA, EMA, S100, BCL2, CD34, and vimentin, were variably expressed, reflecting overlap with other fibroblastic and spindle-cell sarcomas [10]. Tumors with giant rosettes occasionally showed additional positivity for CD68, CD57, and NSE [5,71,72]. Ki-67 indices were low (<10%), supporting the indolent nature of LGFMS.

Given this non-specific profile, MUC4 remains the diagnostic gold standard, with ~100% sensitivity and 70% specificity. In this review, 96% of tested cases were MUC4 positive, confirming its reliability as the most robust immunohistochemical discriminator for LGFMS and related subtypes.

Beyond the canonical FUS– and EWSR1–CREB fusions, a number of rare or novel gene rearrangements and somatic mutations (e.g., t(7;18;16) [73], t(7/10;16) [61], t(4;18) [74], KMT2A-YAP1 [75], KMT2D-PRRX1 [75], HEY1-NCOA2 [42], YAP1-TFE3 [76], MDM2 [77], DDIT3 [77], APC [78], SOX9 [79], TFE3 [79]) have been identified in recent years. Although their clinical significance is unclear, these emerging alterations may refine the molecular classification and prognostic stratification of LGFMS in the future.

### 4.3. Surgical Management and Adjuvant Therapy

Surgical resection remains the cornerstone of curative treatment. Our analysis demonstrated that R0 resections are associated with significantly improved recurrence-free survival and overall survival compared with R1 resections, whereas differences in metastatic risk were not significant. Adjuvant radiotherapy or chemotherapy was rarely administered and showed no proven survival benefit, consistent with prior reports.

The TRASTS phase I–II trial [80], evaluating trabectedin with radiotherapy for advanced soft-tissue sarcomas, reported promising local control but included no LGFMS cases. The potential role of combined (neo)adjuvant regimens therefore remains unproven and should be investigated in prospective, multicenter studies.

### 4.4. Oncological Outcomes and Prognosis

The median follow-up of 3 years in the present review likely underestimates late recurrences and metastases, which can occur decades after surgery, as reflected by the median times to first, local, lung, and other distant recurrence of 3.1, 2.8, 4.0, and 2.3 years, respectively. Local recurrence was observed in 19% of patients and metastasis in 21%, rates consistent with the systematic review by Tang et al. (2010) [1], who reported 29% local and 18% pulmonary recurrence. In contrast, the historic cohort of Evans et al. [45], with follow-up up to 70 years, documented recurrence in 64% and metastasis in 45% of patients, illustrating how extended surveillance dramatically alters perceived outcomes.

In our pooled analysis, 8% of patients died of disease, compared with 3% in Tang’s series and 42% in Evans’. These discrepancies likely reflect shorter observation periods, differences in diagnostic certainty, and earlier inclusion of pathologically unconfirmed cases in older studies.

Overall, the prognosis of LGFMS is generally favorable but unpredictable. Even patients with initially low-grade histology may develop late recurrences or metastases after prolonged disease-free intervals. Since malignant transformation is exceedingly rare and typically symptomatic, a patient-centered, symptom-driven follow-up strategy, with thorough patient education at discharge, is preferred over routine lifelong radiologic surveillance.

### 4.5. Study Limitations

This review is limited by the heterogeneity of included studies, retrospective designs, and variable data completeness—inevitable in rare diseases. Missing data, particularly on follow-up duration and imaging, may have introduced selection and reporting bias. Because of the small number of patients in key subgroups, we were unable to perform reliable comparative analyses for chemotherapy, radiotherapy, or superficial versus deep tumors. Publication bias is also likely, as case reports tend to highlight atypical or aggressive presentations. Efforts were made to minimize duplication by cross-checking large institutional cohorts, but overlapping cases cannot be entirely excluded.

The JBI critical appraisal confirmed overall high methodological quality but recurrent weaknesses in case selection, follow-up completeness, and confounder control.

Despite these limitations, this review represents the largest pooled cohort of LGFMS and subtypes to date, integrating clinical, histopathological, and molecular findings across more than 700 patients.

### 4.6. Clinical Implications

The present analysis underscores the importance of early multidisciplinary evaluation in specialized sarcoma centers, with attention to biopsy quality, margin control, and long-term surveillance. Diagnostic awareness among clinicians and radiologists can substantially reduce inadvertent resections and diagnostic delay.

Given the rarity of LGFMS, international collaboration is essential to establish standardized diagnostic criteria, develop evidence-based follow-up protocols, and explore the role of novel systemic or targeted therapies.

## 5. Conclusions

LGFMS and its subtypes are rare, indolent soft-tissue sarcomas primarily affecting young adults. Although overall survival is favorable, local and distant recurrences remain common and may occur decades after surgery. Wide (R0) resection provides the best local control, whereas (neo)adjuvant treatments lack proven efficacy.

### Recommendations

- Diagnosis should rely on histology and molecular confirmation obtained through core-needle or open biopsy.
- Primary treatment is surgery with the aim of achieving R0 margins.
- Adjuvant therapies may be considered only within clinical trial settings or for unresectable/metastatic disease.
- Follow-up is advised, initially biannually MRI for five years, and then annually MRI up to 10 years, followed by careful patient education at discharge.

Future prospective multicenter studies are required to determine optimal systemic treatment and define the true long-term prognosis of this rare sarcoma spectrum. Long-term effects of these cases are therefore recommended.

## Figures and Tables

**Table 1:** Demographics cohort in median (IQR) and percentage.

	*Median (IQR)* *in Years*	*Total* *Number Described*
*Age*	35 (21–49)	773
*Symptom duration*	1.0 (0.5–4.0)	181
*Follow-up*	3.0 (1.0–6.1)	545
*Time to local recurrence*	2.8 (0.8–6.0)	143
*Time to lung metastasis*	4.0 (0.0–11.3)	62
*Time to metastasis elsewhere*	2.3 (0.0–9.3)	54
	*n* (%)	
*Female (yes)*	389 (50)	773
*Slow growth*	69 (27)	257
*Rapid growth*	24 (9)	257
*Painless*	75 (29)	257
*Painful*	48 (19)	257
*Deep-seated*	475 (80)	598
*MUC4 positive*	243 (57)	428
*Vimentin positive*	198 (46)	428
*Total FUS gene alteration*	263 (83)	318
*FUS/CREB3 positive*	148 (47)	318
*Pathological proven*	472 (61)	773
*Whoops diagnosis*	101 (32)	314
*Surgery*	760 (98)	773
*R0 resection*	279 (66)	423
*R1 resection*	112 (27)	423
*R2 resection*	19 (5)	423
*(Neo)adjuvant chemotherapy*	49 (6)	773
*(Neo)adjuvant radiotherapy*	79 (10)	773
*Complications*	25 (3)	773
*Survival*	501 (92)	545
*Local recurrence*	143 (19)	545
*Lung metastasis*	62 (11)	545
*Metastasis elsewhere*	54 (10)	545

**Table 2:** Pearson chi-square test (*p*-value) and percentage for outcome per resection group.

	*R0* *n (%)*	*R1* *n (%)*	*R0* vs. *R1* *p-Value*
*Survival*	226 (96)	69 (89)	0.02
*Local recurrence*	24 (9)	27 (24)	<0.001
*Lung metastasis*	11 (4)	7 (6)	0.42
*Distant metastasis*	15 (5)	7 (6)	0.81

## Data Availability

The original contributions presented in this study are included in the article/Supplementary Materials. Further inquiries can be directed to the corresponding author.

## Supplementary Materials

The following supporting information can be downloaded at: https://www.mdpi.com/article/10.3390/cancers18030364/s1, Section S1: Figure S1: Representative radiologic images from two institutional cases; Table S1: Overview of institutional cases with clinicopathological characteristics and outcomes (Amsterdam UMC); Section S2: List with included articles; Section S3: Table S2: Summary list with JBI Critical Appraisal Checklist Cohorts ≥ 5 cases; Section S4: Table S3: Pearson chi-square test (*p*-value) and percentage comparing pathology groups proven vs. unproven for outcome per resection group. Supplementary Excel File: Table S4: Tables List with JBI Critical Appraisal Checklist Cohorts ≥ 5 Cases.

## Abbreviations

The following abbreviations are used in this manuscript:

95%-CI	95% Confidence Interval	
AMC	Academic Medical Center	
AWD	Alive with disease	
CNB	Core needle biopsy	
CREB3L1/2	Cyclic AMP responsive element-binding protein 3 like 1 and 2	
CT	Computed Tomography	
DOD	Death of disease	
DOOD	Death of other disease	
DPIA	Data protection impact assessment	
EWSR1	Ewing sarcoma RNA binding protein 1	
FFPE	Formalin-fixed paraffin-embedded	
FISH	Fluorescence in situ hybridization	
FNA	Fine needle aspiration	
FUS	Fused in sarcoma	
HSCTGR	Hyalinizing spindle cell tumor with grand rosettes	
IQR	Interquartile ranges	
LGFMS	Low-grade fibromyxoid sarcoma	
METC	Medical ethics review committee	
MRI	Magnetic Resonance Imaging	
MUC4	Mucin-related antigen 4	
NCBI	National Center for Biotechnology Information	
NED	No evidence of disease	
PET	Positron Emissie Tomografie	
RT-PCR	Reverse transcription-polymerase chain reaction	
SEF	Sclerosing epithelioid fibrosarcoma	
SPSS	Statistical Package for the Social Sciences	
UFMS	Unclassified fibromyxoid sarcoma	
UMC	Amsterdam University Medical Centers	
UvA	University of Amsterdam	
WHO	World Health Organization	
WMO	Medical research involving human subjects act	

## References

[1] Tang Z., Zhou Z.-H., Lv C.-T., Qin L.-Y., Wang Y., Tian G., Luo X.-L., Zhu Q., Xu X.-G. (2010). Low-Grade Fibromyxoid Sarcoma: Clinical Study and Case Report. J. Oral Maxillofac. Surg..

[2] Maretty-Nielsen K., Baerentzen S., Keller J., Dyrop H.B., Safwat A. (2013). Low-Grade Fibromyxoid Sarcoma: Incidence, Treatment Strategy of Metastases, and Clinical Significance of the *FUS* Gene. Sarcoma.

[3] WHO Classification of Tumours Editorial Board (2020). Chapter 1 Soft Tissue Tumours. Soft Tissue and Bone Tumours.

[4] Evans H.L. (1987). Low-grade fibromyxoid sarcoma. A report of two metastasizing neoplasms having a deceptively benign appearance. Am. J. Clin. Pathol..

[5] Lane K.L., Shannon R.J., Weiss S.W. (1997). Hyalinizing spindle cell tumor with giant rosettes: A distinctive tumor closely resembling low-grade fibromyxoid sarcoma. Am. J. Surg. Pathol..

[6] Rekhi B., Deshmukh M., Jambhekar N.A. (2011). Low-grade fibromyxoid sarcoma: A clinicopathologic study of 18 cases, including histopathologic relationship with sclerosing epithelioid fibrosarcoma in a subset of cases. Ann. Diagn. Pathol..

[7] Storlazzi C.T., Mertens F., Nascimento A., Isaksson M., Wejde J., Brosjö O., Mandahl N., Panagopoulos I. (2003). Fusion of the FUS and BBF2H7 genes in low grade fibromyxoid sarcoma. Hum. Mol. Genet..

[8] National Center for Biotechnology Information, U.S. National Library of Medicine. http://www.ncbi.nlm.nih.gov.

[9] GeneCards Human Gene Database GeneCards—Human Genes. Genecards.Org. https://www.genecards.org/.

[10] Guillou L., Benhattar J., Gengler C., Gallagher G., Ranchère-Vince D., Collin F., Terrier P., Terrier-Lacombe M.-J., Leroux A., Marquès B. (2007). Translocation-positive Low-grade Fibromyxoid Sarcoma: Clinicopathologic and Molecular Analysis of a Series Expanding the Morphologic Spectrum and Suggesting Potential Relationship to Sclerosing Epithelioid Fibrosarcoma. Am. J. Surg. Pathol..

[11] Prieto-Granada C., Zhang L., Chen H., Sung Y., Agaram N.P., Jungbluth A.A., Antonescu C.R. (2014). A genetic dichotomy between pure sclerosing epithelioid fibrosarcoma (SEF) and hybrid SEF/low-grade fibromyxoid sarcoma: A pathologic and molecular study of 18 cases. Genes. Chromosom. Cancer.

[12] Hisaoka M., Matsuyama A., Aoki T., Sakamoto A., Yokoyama K. (2012). Low-grade fibromyxoid sarcoma with prominent giant rosettes and heterotopic ossification. Pathol. Res. Pract..

[13] Chan J.K.C. (2013). Newly Available Antibodies With Practical Applications in Surgical Pathology. Int. J. Surg. Pathol..

[14] Domanski H.A., Mertens F., Panagopoulos I., Åkerman M. (2009). Low-grade fibromyxoid sarcoma is difficult to diagnose by fine needle aspiration cytology: A cytomorphological study of eight cases. Cytopathology.

[15] Hwang S., Kelliher E., Hameed M. (2012). Imaging features of low-grade fibromyxoid sarcoma (Evans tumor). Skelet. Radiol..

[16] Yue Y., Liu Y., Song L., Chen X., Wang Y., Wang Z. (2018). MRI findings of low-grade fibromyxoid sarcoma: A case report and literature review. BMC Musculoskelet. Disord..

[17] Chamberlain F., Engelmann B., Al-Muderis O., Messiou C., Thway K., Miah A., Zaidi S., Constantinidou A., Benson C., Gennatas S. (2019). Low-grade Fibromyxoid Sarcoma: Treatment Outcomes and Efficacy of Chemotherapy. In Vivo.

[18] Canpolat C., Evans H.L., Corpron C., Andrassy R.J., Chan K., Eifel P., Elidemir O., Raney B. (1996). Fibromyxoid sarcoma in a four-year-old child: Case report and review of the literature. Med. Pediatr. Oncol..

[19] Arnaud L., Schartz N.E., Bousquet G., Sarandi F., Verola O., Madelaine I., Kerob D., Lebbe C. (2008). Transient Sunitinib-Induced Coma in a Patient With Fibromyxoid Sarcoma. J. Clin. Oncol..

[20] Vandenbroucke J.P., von Elm E., Altman D.G., Gøtzsche P.C., Mulrow C.D., Pocock S.J., Poole C., Schlesselman J.J., Egger M., STROBE Initiative (2007). Strengthening the Reporting of Ob-servational Studies in Epidemiology (STROBE): Explanation and elaboration. Epidemiology.

[21] Page M.J., McKenzie J.E., Bossuyt P.M., Boutron I., Hoffmann T.C., Mulrow C.D., Shamseer L., Tetzlaff J.M., Akl E.A., Brennan S.E. (2021). The PRISMA 2020 statement: An updated guideline for reporting systematic reviews. BMJ.

[22] Munn Z., Barker T.H., Moola S., Tufanaru C., Stern C., McArthur A., Stephenson M., Aromataris M. (2020). Methodological quality of case series studies: An in-troduction to the JBI critical appraisal tool. JBI Evid. Synth..

[23] Ouzzani M., Hammady H., Fedorowicz Z., Elmagarmid A. (2016). Rayyan—A web and mobile app for systematic reviews. Syst. Rev..

[24] Moola S., Munn Z., Tufanaru C., Aromataris E., Sears K., Sfetcu R., Currie M., Lisy K., Qureshi R., Mattis P., Aromataris E., Munn Z. (2020). Chapter 7: Systematic reviews of etiology and risk. JBI Reviewer’s Manual.

[25] Barker T.H., Hasanoff S., Aromataris E., Stone J.C., Leonardi-Bee J., Sears K., Habibi N., Klugar M., Tufanaru C., Moola S. (2024). The revised JBI critical appraisal tool for the assessment of risk of bias for cohort studies. JBI Evid. Synth..

[26] Hermanek P., Wittekind C. (1994). The Pathologist and the Residual Tumor (R) Classification. Pathol. Res. Pract..

[27] Scheer M., Vokuhl C., Veit-Friedrich I., Münter M., von Kalle T., Greulich M., Loff S., Stegmaier S., Sparber-Sauer M., Niggli F. (2019). Low-grade fibromyxoid sarcoma: A report of the Cooperative Weichteilsarkom Studiengruppe (CWS). Pediatr. Blood Cancer.

[28] Gjeorgjievski S.G., Fritchie K., Thangaiah J.J., Folpe A.L., Din N.U. (2021). Head and Neck Low-Grade Fibromyxoid Sarcoma: A Clinicopathologic Study of 15 Cases. Head Neck Pathol..

[29] Geramizadeh B., Zare Z., Dehghanian A.R., Bolandparvaz S., Marzban M. (2018). Huge mesenteric low-grade fibromyxoid sarcoma: A case report and review of the literature. Rare Tumors.

[30] Mulay K., Rao R., Honavar S.G., Reddy V.A.P. (2019). Primary orbital low-grade fibromyxoid sarcoma—A case report. Indian J. Ophthalmol..

[31] Williams C.M., Du W., Mangano W.E., Mei L. (2021). Mediastinal Low-Grade Fibromyxoid Sarcoma With FUS-CREB3L2 Gene Fusion. Cureus.

[32] Lee B.-J., Park W.-S., Jin J.-M., Ha C.-W., Lee S.-H. (2009). Low Grade Fibromyxoid Sarcoma in Thigh. Clin. Orthop. Surg..

[33] Zhang Y., Wan D., Gao F. (2018). Primary low-grade fibromyxoid sarcoma of the breast: A rare case report with immunohistochemical and fluorescence in situ hybridization detection. Hum. Pathol..

[34] Mok Y., Pang Y.H., Sanjeev J.S., Kuick C.H., Chang K.T.-E. (2018). Primary Renal Hybrid Low-grade Fibromyxoid Sarcoma-Sclerosing Epithelioid Fibrosarcoma: An Unusual Pediatric Case With *EWSR1-CREB3L1* Fusion. Pediatr. Dev. Pathol..

[35] Rubinstein J.C., Visa A., Zhang L., Antonescu C.R., Christison-Lagay E.R., Morotti R. (2014). Primary Low-Grade Fibromyxoid Sarcoma of the Kidney in a Child with the Alternative *EWSR1-CREB3L1* Gene Fusion. Pediatr. Dev. Pathol..

[36] Laliberte C., Leong I.T., Holmes H., Monteiro E.A., O’Sullivan B., Dickson B.C. (2017). Sclerosing Epithelioid Fibrosarcoma of the Jaw: Late Recurrence from a Low Grade Fibromyxoid Sarcoma. Head Neck Pathol..

[37] Kesrouani C., Zemoura L., Trassard M., Lae M. (2016). A hybrid lesion: Low-grade fibromyxoid sarcoma (LGFMS) and sclerosing epithelioid fibrosarcoma (SEF). Ann. Pathol..

[38] Kramer S.P., Bowman C.J., Wang Z.J., Sheahon K.M., Nakakura E.K., Cho S.-J., Umetsu S.E., Behr S.C. (2020). Hybrid Low-Grade Fibromyxoid Sarcoma and Sclerosing Epithelioid Fibrosarcoma of the Pancreas. J. Gastrointest. Cancer.

[39] Sharma A., Thangaiah J.J., Shetty S., Policarpio-Nicolas M.L.C. (2021). Bone and soft tissue sarcomas in cerebrospinal fluid and effusion: A 20-year review at our institution. Cancer Cytopathol..

[40] Alfaro-Cervello C., Benavent Casanova O., Nieto G., Mares Diago F.J., Navarro S. (2018). Low-grade fibromyxoid sarcoma, an essential differential diagnosis in myxoid tumors with benign appearance. Rev. Esp. Patol..

[41] Seto T., Song M.-N., Trieu M., Yu J., Sidhu M., Liu C.-M., Sam D., Pan M. (2019). Real-World Experiences with Pazopanib in Patients with Advanced Soft Tissue and Bone Sarcoma in Northern California. Med. Sci..

[42] Murshed K.A., Ammar A. (2020). Hybrid sclerosing epithelioid fibrosarcoma/low grade fibromyxoid sarcoma arising in the small intestine with distinct HEY1-NCOA2 gene fusion. Pathology.

[43] Mustafa S., VandenBussche C.J., Ali S.Z., Siddiqui M.T., Wakely P.E. (2020). Cytomorphologic findings of low-grade fibromyxoid sarcoma. J. Am. Soc. Cytopathol..

[44] Colovic R., Grubor N., Micev M., Jovanovic M., Radak V. (2008). Fibromyxoid sarcoma of the pancreas. Srp. Arh. Celok. Lek..

[45] Evans H.L. (2011). Low-grade fibromyxoid sarcoma: A clinicopathologic study of 33 cases with long-term follow-up. Am. J. Surg. Pathol..

[46] Arbajian E., Puls F., Magnusson L., Thway K., Fisher C., Sumathi V.P., Tayebwa J., Nord K.H., Kindblom L.-G., Mertens F. (2014). Recurrent EWSR1-CREB3L1 Gene Fusions in Sclerosing Epithelioid Fibrosarcoma. Am. J. Surg. Pathol..

[47] Steiner M.A., Giles H.W., Daley W.P. (2009). Massive low-grade fibromyxoid sarcoma presenting as acute respiratory distress in a 12-year-old girl. Pediatr. Radiol..

[48] Alter R.Y., Wamsley C.C., Mullen J.T., Haile W.Z., Goldsmith J.D., Kasper E.M. (2014). Peripheral nerve fibromyxoid sarcoma. J. Neurosurg..

[49] Chetverikova E., Kasenõmm P. (2019). Low-Grade Fibromyxoid Sarcoma of the Lateral Skull Base: Presentation of Two Cases. Case Rep. Otolaryngol..

[50] Deewani M.H., Awan M.S., Din N.U. (2021). Low-grade fibromyxoid sarcoma of the parapharyngeal space: An unusual location. BMJ Case Rep..

[51] Notarnicola A., Moretti L., Cocca M.P., Martucci A., Orsini U., Moretti B. (2010). Low-grade fibromyxoid sarcoma of the medial vastus: A case report. Musculoskelet. Surg..

[52] Hashimoto M., Koide K., Arita M., Kawaguchi K., Mikuriya Y., Iwata J., Iwamoto T. (2016). A Low-Grade Fibromyxoid Sarcoma of the Internal Abdominal Oblique Muscle. Case Rep. Surg..

[53] Ban L.-K., Tseng A.H., Huang S.-H., Lee H.H.-C. (2017). Low-grade fibromyxoid sarcoma of the external anal sphincter: A case report. World J. Surg. Oncol..

[54] Dugalic V., Ignjatovic I.I., Kovac J.D., Ilic N., Sopta J., Ostojic S.R., Vasin D., Bogdanovic M.D., Dumic I., Milovanovic T. (2021). Low-grade fibromyxoid sarcoma of the liver: A case report. World J. Clin. Cases..

[55] Tian K., Johnstone K., Lambie D., Frankel A. (2021). Low-grade fibromyxoid sarcoma with high-grade features, a rare finding. ANZ J. Surg..

[56] Arnaoutoglou C., Lykissas M.G., Gelalis I.D., Batistatou A., Goussia A., Doukas M., Xenakis T.A. (2010). Low grade fibromyxoid sarcoma: A case report and review of the literature. J. Orthop. Surg. Res..

[57] Bajpai J., Shukla S., Jah M., Singh A.K., Goel M., Mourya A., Sachdeva N. (2014). Low-grade fibromyxoid sarcoma around the knee involving the proximal end of the tibia and patella: A rare case report. Oncol. Lett..

[58] Indap S., Dasgupta M., Chakrabarti N., Agarwal A. (2014). Low grade fibromyxoid sarcoma (Evans tumour) of the arm. Indian J. Plast. Surg..

[59] Alatise O., Oke O., Olaofe O., Omoniyi-Esan G., Adesunkanmi A. (2013). A huge low-grade fibromyxoid sarcoma of small bowel mesentery simulating hyper immune splenomegaly syndrome: A case report and review of literature. Afr. Health Sci..

[60] Chitayat S., Barros R., Ribeiro J.G., Silva H.A.M., Sa F.R., Reis B.S.B., Fosse A.M. (2020). Case Report: An extremely rare occurrence of recurrent inguinal low-grade fibromyxoid sarcoma involving the scrotum. F1000Research.

[61] Dobin S.M., Malone V.S., Lopez L., Donner L.R. (2013). Unusual Histologic Variant of a Low-Grade Fibromyxoid Sarcoma in a 3-Year-Old Boy with Complex Chromosomal Translocations Involving 7q34, 10q11.2, and 16p11.2 and Rearrangement of the FUS Gene. Pediatr. Dev. Pathol..

[62] Sedrak M.P., Parker D.C., Gardner J.M. (2013). Low-grade fibromyxoid sarcoma with nuclear pleomorphism arising in the subcutis of a child. J. Cutan. Pathol..

[63] Billings S.D., Giblen G., Fanburg-Smith J.C. (2005). Superficial low-grade fibromyxoid sarcoma (Evans tumor): A clinicopathologic analysis of 19 cases with a unique observation in the pediatric population. Am. J. Surg. Pathol..

[64] Goldstein J.A., Cates J.M. (2015). Differential Diagnostic Considerations of Desmoid-type Fibromatosis. Adv. Anat. Pathol..

[65] Kumari K., Thota R., Chaudhary H.L., Sharma M.C., Thakar A., Singh G. (2019). Low-Grade Fibromyxoid Sarcoma of the External Auditory Canal: A Rare Pathology and Unusual Location. Head Neck Pathol..

[66] Panagopoulos I., Storlazzi C.T., Fletcher C.D., Fletcher J.A., Nascimento A., Domanski H.A., Wejde J., Brosjö O., Rydholm A., Isaksson M. (2004). The chimeric *FUS/CREB3l2* gene is specific for low-grade fibromyxoid sarcoma. Genes Chromosom. Cancer.

[67] Doyle L.A., Möller E., Cin P.D., Fletcher C.D.M., Mertens F., Hornick J.L. (2011). MUC4 Is a Highly Sensitive and Specific Marker for Low-grade Fibromyxoid Sarcoma. Am. J. Surg. Pathol..

[68] Matsuyama A., Hisaoka M., Shimajiri S., Hayashi T., Imamura T., Ishida T., Fukunaga M., Fukuhara T., Minato H., Nakajima T. (2006). Molecular Detection of FUS-CREB3L2 Fusion Transcripts in Low-grade Fibromyxoid Sarcoma Using Formalin-fixed, Paraffin-embedded Tissue Specimens. Am. J. Surg. Pathol..

[69] Lau P.P., Lui P.C., Lau G.T., Yau D.T., Cheung E.T., Chan J.K. (2013). EWSR1-CREB3L1 gene fusion: A novel alternative molecular aberration of low-grade fibromyxoid sarcoma. Am. J. Surg. Pathol..

[70] Vallejo-Benítez A., Rodríguez-Zarco E., Carrasco S.P., Pereira-Gallardo S., Molina J.B., García-Escudero A., Frías A.R., Marcilla D., González-Cámpora R. (2017). Expression of dog1 in low-grade fibromyxoid sarcoma: A study of 19 cases and review of the literature. Ann. Diagn. Pathol..

[71] Scolyer R.A., Mccarthy S.W., Wills E.J., Palmer A.A. (2001). Hyalinising spindle cell tumour with giant rosettes: Report of a case with unusual features including original histological and ultrastructural observations. Pathology.

[72] Bejarano P.A., Padhya T.A., Smith R., Blough R., Devitt J.J., Gluckman J.L. (2000). Hyalinizing spindle cell tumor with giant rosettes—A soft tissue tumor with mesenchymal and neuroendocrine features. An immunohistochemical, ultrastructural, and cytogenetic analysis. Arch. Pathol. Lab. Med..

[73] Wu X., Petrovic V., Torode I.P., Chow C.W. (2009). Low grade fibromyxoid sarcoma: Problems in the diagnosis and management of a malignant tumour with bland histological appearance. Pathology.

[74] Park Y.-H., Kim C.-H., Kim J.-H., Park J.-E., Yim S.-Y. (2018). Rare Concurrence of Congenital Muscular Torticollis and a Malignant Tumor in the Same Sternocleidomastoid Muscle. Ann. Rehabil. Med..

[75] Puls F., Agaimy A., Flucke U., Mentzel T., Sumathi V.P., Ploegmakers M., Stoehr R., Kindblom L.-G., Hansson M., Sydow S. (2020). Recurrent Fusions Between YAP1 and KMT2A in Morphologically Distinct Neoplasms Within the Spectrum of Low-grade Fibromyxoid Sarcoma and Sclerosing Epithelioid Fibrosarcoma. Am. J. Surg. Pathol..

[76] Patton A., Bridge J.A., Liebner D., Chung C., Iwenofu O.H. (2021). A *YAP1::TFE3* cutaneous low-grade fibromyxoid neoplasm: A novel entity!. Genes Chromosomes Cancer.

[77] Huang J., Cohen S., Jour G. (2020). Primary small intestine mesenteric low-grade fibromyxoid sarcoma with foci of atypical epithelioid whorls and diffuse DOG1 expression: A case report. Diagn. Pathol..

[78] Fritch Lilla S.A., Yi J.S., Hall B.A., Moertel C.L. (2014). A novel APC gene mutation associated with a severe phenotype in a patient with Turcot syndrome. J. Pediatr. Hematol. Oncol..

[79] Spalthoff S., Bredt M., Gellrich N.-C., Jehn P. (2016). A Rare Pathology: Low-Grade Fibromyxoid Sarcoma of the Maxilla. J. Oral Maxillofac. Surg..

[80] Martin-Broto J., Hindi N., Lopez-Pousa A., Peinado-Serrano J., Alvarez R., Alvarez-Gonzalez A., Italiano A., Sargos P., Cruz-Jurado J., Isern-Verdum J. (2020). Assessment of Safety and Efficacy of Combined Trabectedin and Low-Dose Radiotherapy for Patients With Metastatic Soft-Tissue Sarcomas: A Non-randomized Phase 1/2 Clinical Trial. JAMA Oncol..

