# Supplementary material for: Low-Grade Fibromyxoid Sarcoma and Related Subtypes: A Systematic Review and Pooled Analysis of 773 Cases
Source: Cancers (Basel). 2026 Jan 23;18(3):364. doi: 10.3390/cancers18030364 (PMC12896792; doi:10.3390/cancers18030364)
Supplement: Supplementary file 1 [file cancers-18-00364-s001.zip › cancers-4074551-Supplementary Sections S1-S4.pdf]

Supplementary Materials

SUPPLEMENTARY MATERIALS S1: Table S1. overview of institutional cases with clinicopathological characteristics and outcomes (Amsterdam UMC).

Case	*Age/Sex*	*Location*	*Size (cm)*	*Histologic subtype / Fusion*	*MUC 4*	*Diagnostic delay (months)*	*Resection margin*	*Adjuvant therapy*	*Follow-up (years)*	*Outcome*
1	8 /M	Abdominal wall	4.0	LGFMS/ FUS-CREB3L2	+	4	R0	None	3.5	NED
2	52 /F	Posterior thigh	12 × 7 × 6	LGFMS/ FUS-CREB3L2	+	0	R0	None	5.1	NED
3	31 /F	Posterolateral knee	8.5 × 6.7 × 2.2	LGFMS/ FUS-CREB3L2	+	18	R1 (< 1 mm)	None	3.1	NED
4	76 /M	Distal thigh	11.5	LGFMS/ FUS-CREB3L2	+	-	R1	None	1.7	NED

Abbreviations: LGFMS, low-grade fibromyxoid sarcoma; UFMS, unclassified fibromyxoid sarcoma; NED, no evidence of disease.

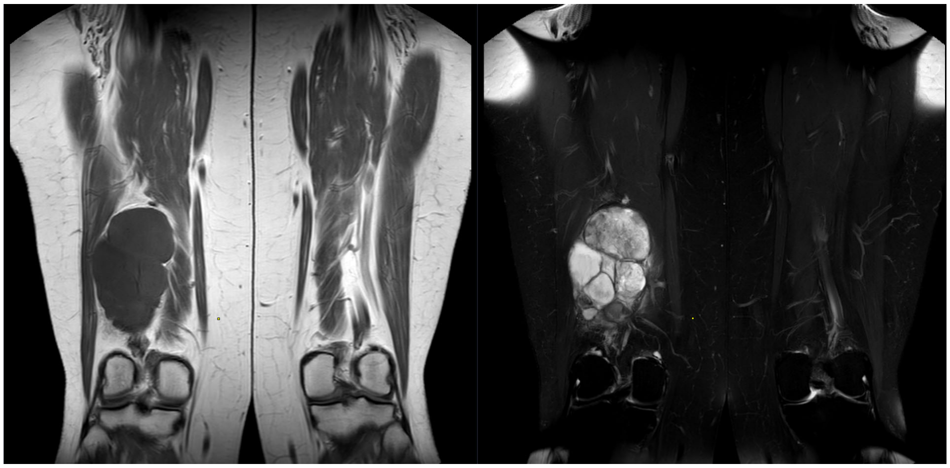

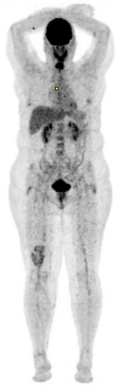

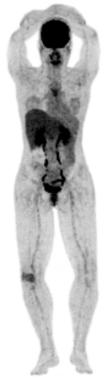

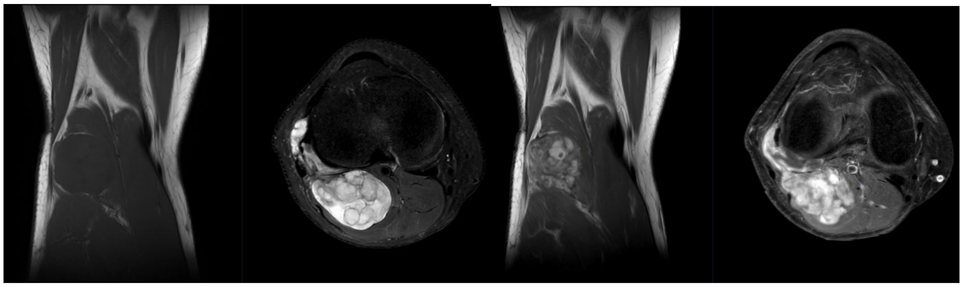

Figure S1. Representative radiologic images from two institutional cases

All four institutional cases had a deep-seated intramuscular or subfascial lesions, well-circumscribed on MRI, with a mixed fibrous (T1/T2 low) and myxoid (T2 high) signal pattern. Post-contrast enhancement was heterogeneous with a gyriform configuration. FDG-PET/CT demonstrated low-to-moderate metabolic activity in all cases (SUVmax ≤ 4.5).

Supplementary Materials S2: List with included articles

1. Evans (1987)(1)
2. Devaney (1990)(2)
3. Paulus (1991)(3)
4. Evans (1993)(4)
5. Nichols (1994)(5)
6. Ugai (1994)(6)
7. Goodlad (1995)(7)
8. Fukunaga (1996)(8)
9. Canpolat (1996)(9)
10. Papadimitriou (1997)(10)
11. Resnick (1997)(11)
12. Dvornik (1997)(12)
13. Lane (1997)(13)
14. Ludvíková (1998)(14)
15. Magro (1998)(15)
16. Shidham (1999)(16)
17. Lindberg (1999)(17)
18. Nielsen (1999)(18)
19. Takanami (1999)(19)
20. Meister (1999)(20)
21. Woodruff (1999)(21)
22. Van den Bossche (2000)(22)
23. Zámecník (2000)(23)
24. Silverman (2000)(24)
25. Mezzelani (2000)(25)
26. Farinha (2000)(26)
27. Bejarano (2000)(27)
28. Scolyer (2001)(28)
29. Fras (2001)(29)
30. Dobashi (2001)(30)
31. O'Sullivan (2002)(31)
32. Storlazzi (2003)(32)
33. Reid (2003)(33)
34. Harish (2003)(34)
35. Koishi (2003)(35)
36. Yang (2003)(36)
37. Estrada-Villaseñor (2004)(37)
38. Mountney (2004)(38)
39. Panagopoulos (2004)(39)
40. Galetta (2004)(40)
41. Oda (2004)(41)
42. Chang (2004)(42)
43. Williams (2004)(43)
44. Koh (2005)(44)
45. Mertens (2005)(45)
46. Billings (2005)(46)
47. Rando (2005)(47)
48. Antonescu (2004)(48)
49. Bhattacharya (2005)(49)
50. Kim (2005)(50)
51. Kusumi (2005)(51)
52. Park (2007)(52)
53. Dawamneh (2006)(53)
54. Torriani (2006)(54)
55. Matsuyama (2006)(55)
56. Miyake (2006)(56)
57. Botev (2006)(57)
58. Périgny (2006)(58)
59. Kim (2007)(59)
60. Winfield (2007)(60)
61. Guillou (2007)(61)
62. Vlacha (2008)(62)
63. Jannasch (2008)(63)
64. Jakowski (2008)(64)
65. Arnaud (2008)(65)
66. Saito (2008)(66)
67. Fujii (2008)(67)
68. Shahdadpuri (2008)(68)
69. Domanski (2009)(69)
70. Colović (2008)(70)
71. Tun (2008)(71)
72. Kalebi (2008)(72)
73. Meng (2009)(73)
74. Guenther (2008)(74)
75. Wu (2009)(75)
76. Steiner (2009)(76)
77. Thway (2009)(77)
78. Liao (2009)(78)
79. Del Valle González (2009)(79)
80. Maeda (2009)(80)
81. Lee (2009)(81)
82. Merchant (2009)(82)
83. Kim (2010)(83)
84. Tang (2010)(84)
85. Notarnicola (2010)(85)
86. Bartuma (2010)(86)
87. Dhillon (2010)(87)

88. Wollina (2010)(88)
89. Higuchi (2010)(89)
90. Ballesteros Gomiz (2011)(90)
91. Arnaoutoglou (2010)(91)
92. Ramaswamy (2011)(92)
93. Baydar (2010)(93)
94. Arancio (2010)(94)
95. Lee (2010)(95)
96. Kaoutar (2011)(96)
97. Manes (2011)(97)
98. Möller (2011)(98)
99. Rekhi (2011)(99)
100. Lee (2011)(100)
101. Laurini (2011)(101)
102. Kim (2011)(102)
103. Evans (2011)(103)
104. Menon (2012)(104)
105. Rubin (2010)(105)
106. Brasanac (2013)(106)
107. Lam (2012)(107)
108. Barnhill (2012)(108)
109. Hisaoka (2012)(109)
110. Abe (2012)(110)
111. Dobin (2013)(111)
112. Odem (2013)(112)
113. Alevizopoulos (2012)(113)
114. VanSandt (2013)(114)
115. Ferlosio (2013)(115)
116. Dong (2013)(116)
117. He (2013)(117)
118. Tominaga (2014)(118)
119. Lau (2013)(119)
120. Bhandarkar (2013)(120)
121. Furudate (2013)(121)
122. Maretty-Nielsen (2013)(122)
123. Singh (2012)(123)
124. Ferreira (2013)(124)
125. Sedrak (2014)(125)
126. Alatise (2013)(126)
127. Fritch Lilla (2014)(127)
128. Arbajian (2014)(128)
129. Thapar (2014)(129)
130. Kim (2014)
131. Linos (2014)(131)
132. Alter (2014)(132)
133. Sugita (2014)(133)
134. Rubinstein (2014)(134)
135. Wojcik (2014)(135)
136. Bajpai (2014)(136)
137. Whale (2014)(137)
138. Liang (2014)(138)
139. Citores-Pascual (2013)(139)
140. Güdücü (2014)(140)
141. Kurisaki-Arakawa (2014)(141)
142. Indap (2014)(142)
143. Prieto-Granada (2015)(143)
144. Papp (2015)(144)
145. Unlü (2015)(145)
146. Kurisaki-Arakawa (2014)(146)
147.Thway (2015)(147)
148. Mendoza (2015)(148)
149. Chen (2015)(149)
150. Varsak (2015)(150)
151. Goldstein (2015)(151)
152. Thway (2015)(152)
153. White (2015)(153)
154. Sargar (2015)(154)
155. Konecna (2015)(155)
156. Cowan (2016)(156)
157. Lee (2015)(157)
158. Spalthoff (2016)(158)
159. Benhiba (2015)(159)
160. Soma (2015)(160)
161. Mazari (2015)(161)
162. Lee (2016)(162)
163. Zou (2016)(163)
164. Chien (2016)(164)
165. Shibata (2016)(165)
166. Chatterjee (2016)(166)
167. Mastoraki (2015)(167)
168. Hashimoto (2016)(168)
169. Chaudhuri (2016)(169)
170. Kesrouani (2016)(170)
171. Sung (2017)(171)
172. Ban (2017)(172)
173. Mentzel (2017)(173)
174. Vallejo-Benítez (2017)(174)
175. Rakheja (2017)(175)
176. Tan (2017)(176)
177. Laliberte (2018)(177)
178. Swanson (2018)(178)
179. Ud Din (2018)(179)
180. Wang (2017)(180)
181. Mok (2018)(181)
182. Yue (2018)(182)
183. Zhang (2018)(183)
184. Tay (2018)(184)
185. Park (2018)(185)
186. Geramizadeh (2018)(186)
187. Bhattar (2017)(187)
188. Alfaro-Cervelló (2017)(188)
189. Han (2018)(189)
190. Karthigeyan (2019)(190)
191. Sambri (2018)(191)
192. Saab-Chalhoub (2019)(192)
193. Seto (2019)(193)
194. Rao (2019)(194)
195. Kumari (2020)(195)
196. Chetverikova (2019)(196)
197. Scheer (2020)(197)
198. Kanato (2019)(198)
199. Fuentes (2020)(199)
200. Shimizu (2020)(200)
201. Puls (2020)(201)
202. Zhao (2020)(202)
203. Kramer (2020)(203)
204. Huang (2020)(204)
205. Mustafa (2020)(205)
206. Logan (2020)(206)

207. Murshed (2020)(207)
208. Koucky (2021)(208)
209. Perez (2020)(209)
210. Yuan (2020)(210)
211. Chitayat (2020)(211)
212. Kosemehmetoglu (2021)(212)
213. Righi (2021)(213)
214. Dugalic (2021)(214)
215. Oramas (2021)(215)
216. Sajid (2021)(216)
217. Auria-Aspiazu (2021)(217)
218. Kiyohara (2020)(218)
219. Deewani (2021)(219)
220. Niepokój-Czopnik (2021)(220)
221. Chivchibashi (2021)(221)
222. Sharma (2021)(222)
223. Williams (2021)(223)
224. Yoshimura (2021)(224)
225. Naik (2021)(225)
226. Yoshimatsu (2021)(226)
227. Gjorgova Gjeorgjevski (2021)(227)
228. Cantu (2021)(228)
229. Tian (2021)(229)
230. Patton (2021)(230)
231. Zhang (2022)(231)
232. Froiio (2022)(232)
233. Chan (2022)(233)
234. Nunes Pombo (2022)(234)
235. Khamashta (2022)(235)
236. Costigan (2022)(236)
237. Guo (2022)(237)
238. Xie (2022)(238)
239. Ershadi (2022)(239)
240. Sahraoui (2022)(240)
241. Tan(2022)(241)
242. Ronen (2023)(242)
243. He (2023)(243)
244. Muñoz-Leija (2023)(244)
245. Lavin (2023)(245)
246. Weiler (2023)(246)
247. Narukami (2023)(247)
248. Otieno (2023)(248)
249. Theodorou (2023)(249)
250. Cloutier (2023)(250)
251. Basiri (2023)(251)
252. Nawojowska (2023)(252)
253. Tasaki (2023)(253)
254. Anehosur (2023)(254)
255. Johnson (2024)(255)
256. Ayadi (2024)(256)
257. Zhang (2024)(257)
258. Watanabe (2024)(258)
259. Ishida (2024)(259)
260. Suster (2024)(260)
261. Alkameshki (2024)(261)
262. Tally (2024)(262)
263. Barger (2024)(263)
264. Noguchi (2024)(264)
265. Quiceno (2024)(265)
266. Oh (2024)(266)
267. Fernández (2024)(267)
268. Suleman (2024)(268)
269. Jia (2025)(269)
270. Salinas-Castro (2025)(270)
271. Kubetsky (2025)(271)
272. Kinoshita (2025)(272)
273. Mohamedali (2025)(273)

SUPPLEMENTARY MATERIALS S3:

TABLE S2. Summary list with JBI critical appraisal checklist Cohorts ≥ 5 cases.

JBI-criterium	*Yes (n)*	*Yes (%)*	*Unclear (n)*	*Unclear (%)*	*No (n)*	*No (%)*
1.	29	97 %	1	3 %	0	0 %
2.	28	93 %	2	7 %	0	0 %
3.	28	93 %	1	3 %	1	3 %
4.	22	73 %	6	20 %	2	7 %
5.	23	77 %	5	17 %	2	7 %
6.	27	90 %	3	10 %	0	0 %
7.	27	90 %	2	7 %	1	3 %
8.	26	87 %	3	10 %	1	3 %
9.	25	83 %	3	10 %	2	7 %
10.	24	80 %	4	13 %	2	7 %
11.	28	93 %	2	7 %	0	0 %

SUPPLEMENTARY MATERIALS S4

TABLE S3. Pearson chi-square test (*p*-value) and percentage comparing pathology groups proven vs. unproven for outcome per resection group

	R0 proven	*R0 unproven*	*P-value*	*R1 proven*	*R1 unproven*	*p-value*
Survival	95%	97%	0.57	89%	89%	0.85
Local recurrence	9%	8%	0.86	26%	22%	0.61
Lung metastasis	5%	3%	0.45	7%	5%	0.64
Distant metastasis	7%	4%	0.26	3%	9%	0.21

References

1. Evans HL. Low-grade fibromyxoid sarcoma. A report of two metastasizing neoplasms having a deceptively benign appearance. Am J Clin Pathol. 1987;88(5):615–9.
2. Devaney DM, Dervan P, O'Neill S, Carney D, Leader M. Low-grade fibromyxoid sarcoma. Histopathology. 1990;17(5):463–5.
3. Paulus W, Slowik F, Jellinger K. Primary intracranial sarcomas: histopathological features of 19 cases. Histopathology. 1991;18(5):395–402.
4. Evans HL. Low-grade fibromyxoid sarcoma. A report of 12 cases. Am J Surg Pathol. 1993;17(6):595–600.
5. Nichols GE, Cooper PH. Low-grade fibromyxoid sarcoma: case report and immunohistochemical study. J Cutan Pathol. 1994;21(4):356–62.
6. Ugai K, Kizaki T, Morimoto K, Sashikata T. A case of low-grade fibromyxoid sarcoma of the thigh. Pathol Int. 1994;44(10-11):793–9.
7. Goodlad JR, Mentzel T, Fletcher CD. Low grade fibromyxoid sarcoma: clinicopathological analysis of eleven new cases in support of a distinct entity. Histopathology. 1995;26(3):229–37.
8. Fukunaga M, Ushigome S, Fukunaga N. Low-grade fibromyxoid sarcoma. Virchows Arch. 1996;429(4-5):301–3.
9. Canpolat C, Evans HL, Corpron C, Andrassy RJ, Chan K, Eifel P, et al. Fibromyxoid sarcoma in a four-year-old child: case report and review of the literature. Med Pediatr Oncol. 1996;27(6):561–4.
10. Papadimitriou JC, Ord RA, Drachenberg CB. Head and neck fibromyxoid sarcoma: clinicopathological correlation with emphasis on peculiar ultrastructural features related to collagen processing. Ultrastruct Pathol. 1997;21(1):81–7.
11. Resnick JM, Fanning CV, Caraway NP, Varma DG, Johnson M. Percutaneous needle biopsy diagnosis of benign neurogenic neoplasms. Diagn Cytopathol. 1997;16(1):17–25.
12. Dvornik G, Barbareschi M, Gallotta P, Dalla Palma P. Low grade fibromyxoid sarcoma. Histopathology. 1997;30(3):274–6.
13. Lane KL, Shannon RJ, Weiss SW. Hyalinizing spindle cell tumor with giant rosettes: a distinctive tumor closely resembling low-grade fibromyxoid sarcoma. Am J Surg Pathol. 1997;21(12):1481–8.
14. Ludvikova M, Michal M, Zamecnik M. Hyalinizing spindle cell tumors with giant rosette-like structures. Pathol Res Pract. 1998;194(8):577–81; discussion 83–6.
15. Magro G, Fraggetta F, Manusia M, Mingrino A. Hyalinizing spindle cell tumor with giant rosettes: a previously undescribed lesion of the lung. Am J Surg Pathol. 1998;22(11):1431–3.
16. Shidham VB, Ayala GE, Lahaniatis JE, Garcia FU. Low-grade fibromyxoid sarcoma: clinicopathologic case report with review of the literature. Am J Clin Oncol. 1999;22(2):150–5.
17. Lindberg GM, Maitra A, Gokaslan ST, Saboorian MH, Albores-Saavedra J. Low grade fibromyxoid sarcoma: fine-needle aspiration cytology with histologic, cytogenetic, immunohistochemical, and ultrastructural correlation. Cancer. 1999;87(2):75–82.
18. Nielsen GP, Selig MK, O'Connell JX, Keel SB, Dickersin GR, Rosenberg AE. Hyalinizing spindle cell tumor with giant rosettes: a report of three cases with ultrastructural analysis. Am J Surg Pathol. 1999;23(10):1227–32.
19. Takanami I, Takeuchi K, Naruke M. Low-grade fibromyxoid sarcoma arising in the mediastinum. J Thorac Cardiovasc Surg. 1999;118(5):970–1.
20. Meister P, Babaryka I. [Hyalinizing spindle cell tumor with giant rosettes. Case report with immunohistochemical characterization]. Pathologe. 1999;20(3):183–8.
21. Woodruff JM, Antonescu CR, Erlandson RA, Boland PJ. Low-grade fibrosarcoma with palisaded granulomalike bodies (giant rosettes): report of a case that metastasized. Am J Surg Pathol. 1999;23(11):1423–8.
22. van den Bossche MR, Van Mieghem H. Low-grade fibromyxoid sarcoma. Oncology. 2000;58(3):207–9.

23. Zamecnik M, Michal M. Low-grade fibromyxoid sarcoma: a report of eight cases with histologic, immunohistochemical, and ultrastructural study. Ann Diagn Pathol. 2000;4(4):207–17.
24. Silverman JF, Nathan G, Olson PR, Prichard J, Cohen JK. Fine-needle aspiration cytology of low-grade fibromyxoid sarcoma of the renal capsule (capsuloma). Diagn Cytopathol. 2000;23(4):279–83.
25. Mezzelani A, Sozzi G, Nessling M, Riva C, Della Torre G, Testi MA, et al. Low grade fibromyxoid sarcoma. a further low-grade soft tissue malignancy characterized by a ring chromosome. Cancer Genet Cytogenet. 2000;122(2):144–8.
26. Farinha P, Oliveira P, Soares J. Metastasizing hyalinizing spindle cell tumour with giant rosettes: report of a case with long survival. Histopathology. 2000;36(1):92–3.
27. Bejarano PA, Padhya TA, Smith R, Blough R, Devitt JJ, Gluckman JL. Hyalinizing spindle cell tumor with giant rosettes--a soft tissue tumor with mesenchymal and neuroendocrine features. An immunohistochemical, ultrastructural, and cytogenetic analysis. Arch Pathol Lab Med. 2000;124(8):1179–84.
28. Scolyer RA, McCarthy SW, Wills EJ, Palmer AA. Hyalinising spindle cell tumour with giant rosettes: report of a case with unusual features including original histological and ultrastructural observations. Pathology. 2001;33(1):101–7.
29. Fras AP, Frkovic-Grazio S. Hyalinizing spindle cell tumor with giant rosettes of the broad ligament. Gynecol Oncol. 2001;83(2):405–8.
30. Dobashi Y, Noguchi T, Nasuno S, Jiang SX, Kameya T. Hyalinizing spindle cell tumor with giant rosettes: report of a case showing remarkable myofibroblastic differentiation. Pathol Res Pract. 2001;197(10):691–7.
31. O'Sullivan MJ, Sirgi KE, Dehner LP. Low-grade fibrosarcoma (hyalinizing spindle cell tumor with giant rosettes) with pulmonary metastases at presentation: case report and review of the literature. Int J Surg Pathol. 2002;10(3):211–6.
32. Storlazzi CT, Mertens F, Nascimento A, Isaksson M, Wejde J, Brosjo O, et al. Fusion of the FUS and BBF2H7 genes in low grade fibromyxoid sarcoma. Hum Mol Genet. 2003;12(18):2349–58.
33. Reid R, de Silva MV, Paterson L, Ryan E, Fisher C. Low-grade fibromyxoid sarcoma and hyalinizing spindle cell tumor with giant rosettes share a common t(7;16)(q34;p11) translocation. Am J Surg Pathol. 2003;27(9):1229–36.
34. Harish K, Ashok AC, Alva NK. Low grade fibromyxoid sarcoma of the falciform ligament: a case report. BMC Surg. 2003;3:7.
35. Koishi A, Gomibuchi H, Inoue J, Minoura S, Itoh E, Saito M. Hyalinizing spindle cell tumor with giant rosettes of the omentum. J Obstet Gynaecol Res. 2003;29(6):388–91.
36. Yang SF, Liu CS, Chai CY, Chien SH, Wang CK. Hyalinizing spindle cell tumor with giant rosettes: a case report. Kaohsiung J Med Sci. 2003;19(11):583–7.
37. Estrada Villasenor EG, Delgado Cedillo EA, Linares Gonzalez LM, Rico Martinez G. Fine needle aspiration cytology of low grade fibromyxoid sarcoma. Report of a case with histologic correlation. Acta Cytol. 2004;48(1):69–72.
38. Mountney J, Thomas NP. When is a meniscal cyst not a meniscal cyst? Knee. 2004;11(2):133–6.
39. Panagopoulos I, Storlazzi CT, Fletcher CD, Fletcher JA, Nascimento A, Domanski HA, et al. The chimeric FUS/CREB3l2 gene is specific for low-grade fibromyxoid sarcoma. Genes Chromosomes Cancer. 2004;40(3):218–28.
40. Galetta D, Cesario A, Margaritora S, Granone P. Primary mediastinal hyalinizing spindle cell tumor with giant rosettes. Ann Thorac Surg. 2004;77(6):2206–9.
41. Oda Y, Takahira T, Kawaguchi K, Yamamoto H, Tamiya S, Matsuda S, et al. Low-grade fibromyxoid sarcoma versus low-grade myxofibrosarcoma in the extremities and trunk. A comparison of clinicopathological and immunohistochemical features. Histopathology. 2004;45(1):29–38.
42. Chang E, Lee A, Lee E, Shin O, Kang C, Kim JM, et al. Hyalinizing spindle cell tumor with giant rosettes with pulmonary metastasis after a long hiatus: a case report. J Korean Med Sci. 2004;19(4):619–23.
43. Williams HT, Gossage JR, Jr., Allred TJ, Kallab AM, Pancholy A, Anstadt MP. F-18 FDG positron emission tomography imaging of rare soft tissue sarcomas: low-grade fibromyxoid sarcoma and malignant hemangiopericytoma. Clin Nucl Med. 2004;29(9):581–4.

44. Koh SH, Choe HS, Lee IJ, Park HR, Bae SH. Low-grade fibromyxoid sarcoma: ultrasound and magnetic resonance findings in two cases. Skeletal Radiol. 2005;34(9):550–4.
45. Mertens F, Fletcher CD, Antonescu CR, Coindre JM, Colecchia M, Domanski HA, et al. Clinicopathologic and molecular genetic characterization of low-grade fibromyxoid sarcoma, and cloning of a novel FUS/CREB3L1 fusion gene. Lab Invest. 2005;85(3):408–15.
46. Billings SD, Giblen G, Fanburg-Smith JC. Superficial low-grade fibromyxoid sarcoma (Evans tumor): a clinicopathologic analysis of 19 cases with a unique observation in the pediatric population. Am J Surg Pathol. 2005;29(2):204–10.
47. Rando G, Buonuomo V, D'Urzo C, Vecchio F, Caldarelli M, Pintus C. Fibromyxoid sarcoma in a 4-year-old boy: case report and review of the literature. Pediatr Surg Int. 2005;21(4):311–2.
48. Antonescu CR, Baren A. Spectrum of low-grade fibrosarcomas: a comparative ultrastructural analysis of low-grade myxofibrosarcoma and fibromyxoid sarcoma. Ultrastruct Pathol. 2004;28(5-6):321–32.
49. Bhattacharya B, Dilworth HP, Iacobuzio-Donahue C, Ricci F, Weber K, Furlong MA, et al. Nuclear beta-catenin expression distinguishes deep fibromatosis from other benign and malignant fibroblastic and myofibroblastic lesions. Am J Surg Pathol. 2005;29(5):653–9.
50. Kim SY, Kim MY, Hwang YJ, Han YH, Seo JW, Kim YH, et al. Low-grade fibromyxoid sarcoma: CT, sonography, and MR findings in 3 cases. J Thorac Imaging. 2005;20(4):294–7.
51. Kusumi T, Nishikawa S, Tanaka M, Ogawa T, Jin H, Sato F, et al. Low-grade fibromyxoid sarcoma arising in the big toe. Pathol Int. 2005;55(12):802–6.
52. Park IJ, Kim HC, Yu CS, Kim JS, Jang SJ, Kim JC. Low-grade fibromyxoid sarcoma of the colon. Dig Liver Dis. 2007;39(3):274–7.
53. Dawamneh MF, Amra NK, Amr SS. Low grade fibromyxoid sarcoma: report of a case with fine needle aspiration cytology and histologic correlation. Acta Cytol. 2006;50(2):208–12.
54. Torriani M, Etchebehere M, Amstalden EM, Ouellette H. Magnetic resonance imaging of low-grade fibromyxoid sarcoma. Clinics (Sao Paulo). 2006;61(3):267–70.
55. Matsuyama A, Hisaoka M, Shimajiri S, Hayashi T, Imamura T, Ishida T, et al. Molecular detection of FUS-CREB3L2 fusion transcripts in low-grade fibromyxoid sarcoma using formalin-fixed, paraffin-embedded tissue specimens. Am J Surg Pathol. 2006;30(9):1077–84.
56. Miyake M, Tateishi U, Maeda T, Arai Y, Seki K, Hasegawa T, et al. CT and MRI features of low-grade fibromyxoid sarcoma in the shoulder of a pediatric patient. Radiat Med. 2006;24(7):511–4.
57. Botev B, Casale M, Vincenzi B, D'Ascanio L, Santini D, Esposito V, et al. A giant sarcoma of the parotid gland: a case report and review of the literature. In Vivo. 2006;20(6B):907–10.
58. Perigny M, Dion N, Couture C, Lagace R. [Low grade fibromyxoid sarcoma: a clinico-pathologic analysis of 7 cases]. Ann Pathol. 2006;26(6):419–25.
59. Kim L, Yoon YH, Choi SJ, Han JY, Park IS, Kim JM, et al. Hyalinizing spindle cell tumor with giant rosettes arising in the lung: report of a case with FUS-CREB3L2 fusion transcripts. Pathol Int. 2007;57(3):153–7.
60. Winfield HL, De Las Casas LE, Greenfield WW, Santin AD, McKenney JK. Low-grade fibromyxoid sarcoma presenting clinically as a primary ovarian neoplasm: a case report. Int J Gynecol Pathol. 2007;26(2):173–6.
61. Guillou L, Benhattar J, Gengler C, Gallagher G, Ranchere-Vince D, Collin F, et al. Translocation-positive low-grade fibromyxoid sarcoma: clinicopathologic and molecular analysis of a series expanding the morphologic spectrum and suggesting potential relationship to sclerosing epithelioid fibrosarcoma: a study from the French Sarcoma Group. Am J Surg Pathol. 2007;31(9):1387–402.
62. Vlacha V, Repanti M. Low grade fibromyxoid sarcoma and neurofibromatosis type I. Pediatr Blood Cancer. 2008;50(4):938.
63. Jannasch O, Dombrowski F, Lippert H, Meyer F. Rare coincidence of familial adenomatous polyposis and a retroperitoneal fibromyxoid sarcoma: report of a case. Dis Colon Rectum. 2008;51(4):477–81.
64. Jakowski JD, Wakely PE, Jr. Primary intrathoracic low-grade fibromyxoid sarcoma. Hum Pathol. 2008;39(4):623–8.
65. Arnaud L, Schartz NE, Bousquet G, Sarandi F, Verola O, Madelaine I, et al. Transient sunitinib-induced coma in a patient with fibromyxoid sarcoma. J Clin Oncol. 2008;26(9):1569–71.

66. Saito R, Kumabe T, Watanabe M, Jokura H, Shibuya M, Nakazato Y, et al. Low-grade fibromyxoid sarcoma of intracranial origin. J Neurosurg. 2008;108(4):798–802.
67. Fujii S, Kawawa Y, Horiguchi S, Kamata N, Kinoshita T, Ogawa T. Low-grade fibromyxoid sarcoma of the small bowel mesentery: computed tomography and magnetic resonance imaging findings. Radiat Med. 2008;26(4):244–7.
68. Shahdadpuri R, O'Meara A, O'Sullivan M, Reardon W. Low-grade fibromyxoid sarcoma: yet another malignancy associated with Kabuki syndrome. Clin Dysmorphol. 2008;17(3):199–202.
69. Domanski HA, Mertens F, Panagopoulos I, Akerman M. Low-grade fibromyxoid sarcoma is difficult to diagnose by fine needle aspiration cytology: a cytomorphological study of eight cases. Cytopathology. 2009;20(5):304–14.
70. Colovic R, Grubor N, Misev M, Jovanovic M, Radak V. [Fibromyxoid sarcoma of the pancreas]. Srp Arh Celok Lek. 2008;136(3-4):158–61.
71. Tun K, Ozen O, Kaptanoglu E, Gurcan O, Beskonakli E, Celasun B. Primary intracranial low-grade fibromyxoid sarcoma (Evans tumor). J Clin Neurosci. 2008;15(11):1298–301.
72. Kalebi AY, Hale MJ. Pulmonary metastasis from a deltoid subcutaneous low-grade fibromyxoid sarcoma with giant collagen rosettes. Hum Pathol. 2008;39(10):1553–4; author reply 4.
73. Meng GZ, Zhang HY, Bu H, Geng JG. Low-grade fibromyxoid sarcoma versus fibromatosis: a comparative study of clinicopathological and immunohistochemical features. Diagn Cytopathol. 2009;37(2):96–102.
74. Guenther N, Menenakos C, Braumann C, Buettemeyer R. Hyalinizing spindle cell tumor with giant rosettes of the hand. A surgical and histopathological rarity. Acta Chir Belg. 2008;108(5):610–2.
75. Wu X, Petrovic V, Torode IP, Chow CW. Low grade fibromyxoid sarcoma: problems in the diagnosis and management of a malignant tumour with bland histological appearance. Pathology. 2009;41(2):155–60.
76. Steiner MA, Giles HW, Daley WP. Massive low-grade fibromyxoid sarcoma presenting as acute respiratory distress in a 12-year-old girl. Pediatr Radiol. 2009;39(4):396–9.
77. Thway K, Fisher C, Debiec-Rychter M, Calonje E. Claudin-1 is expressed in perineurioma-like low-grade fibromyxoid sarcoma. Hum Pathol. 2009;40(11):1586–90.
78. Liao KS, Huang WT, Yang SF, Chien SH, Hsieh TJ, Chai CY, et al. Intramuscular low-grade fibromyxoid sarcoma: a case report. Kaohsiung J Med Sci. 2009;25(8):448–54.
79. del Valle Gonzalez N, Santos Largo J, Martinez-Sagarra Oceja J, Rodriguez Tesedo V. [Fibromyxoid sarcoma of kidney]. Actas Urol Esp. 2009;33(9):1036–9.
80. Maeda E, Ohta S, Watadani T, Goto A, Nakajima A, Ohtomo K. Imaging findings of thoracic low-grade fibromyxoid sarcoma: report of three cases. Jpn J Radiol. 2009;27(9):375–80.
81. Lee BJ, Park WS, Jin JM, Ha CW, Lee SH. Low grade fibromyxoid sarcoma in thigh. Clin Orthop Surg. 2009;1(4):240–3.
82. Merchant SH. Low grade fibromyxoid sarcoma: report of a case with epithelioid cell morphology, masquerading as a papillary thyroid carcinoma. Acta Cytol. 2009;53(6):689–92.
83. Kim UR, Arora V, Ramchandran S, Shah AD, Phelps PO. Orbital hyalinizing spindle cell tumor with giant rosettes. Ophthalmic Plast Reconstr Surg. 2010;26(1):30–2.
84. Tang Z, Zhou ZH, Lv CT, Qin LY, Wang Y, Tian G, et al. Low-grade fibromyxoid sarcoma: clinical study and case report. J Oral Maxillofac Surg. 2010;68(4):873–84.
85. Notarnicola A, Moretti L, Cocca MP, Martucci A, Orsini U, Moretti B. Low-grade fibromyxoid sarcoma of the medial vastus: a case report. Musculoskelet Surg. 2010;94(2):109–12.
86. Bartuma H, Moller E, Collin A, Domanski HA, Von Steyern FV, Mandahl N, et al. Fusion of the FUS and CREB3L2 genes in a supernumerary ring chromosome in low-grade fibromyxoid sarcoma. Cancer Genet Cytogenet. 2010;199(2):143–6.
87. Dhillon MS, Mootha AK, Kumar V, Saini R, Bharti S. Low-grade fibromyxoid sarcoma of the talus: a case report. J Foot Ankle Surg. 2010;49(4):400 e5–8.
88. Wollina U, Runge J, Schonlebe J. Fibromyxoid sarcoma of the leg. Indian Dermatol Online J. 2010;1(1):24–6.
89. Higuchi M, Suzuki H, Shio Y, Hoshi S, Gotoh M. Successfully resected intrathoracic low-grade fibromyxoid sarcoma. Gen Thorac Cardiovasc Surg. 2010;58(7):348–51.

90. Ballesteros Gomiz E, Casalots Casado J, Rovira Gols A, Prenafeta Moreno M. [Low-grade intracranial fibromyxoid sarcoma: A case report]. Radiologia. 2011;53(2):171–4.
91. Arnaoutoglou C, Lykissas MG, Gelalis ID, Batistatou A, Goussia A, Doukas M, et al. Low grade fibromyxoid sarcoma: a case report and review of the literature. J Orthop Surg Res. 2010;5:49.
92. Ramaswamy AS, Chatura KR. Hyalinizing spindle cell tumor with giant rosettes. Ann Saudi Med. 2011;31(1):83–6.
93. Baydar DE, Aki FT. Low-grade fibromyxoid sarcoma metastatic to the prostate. Ann Diagn Pathol. 2011;15(1):64–8.
94. Arancio M, Ranzoni S, Delsignore A, Maffei N, Landi G, Mina A, et al. [Fibromyxoid sarcoma of the kidney. A case report]. Urologia. 2010;77(4):263–6.
95. Lee WJ, Park CO, Yoon SH, Chu YC. Primary paravertebral low-grade fibromyxoid sarcoma. J Korean Neurosurg Soc. 2010;48(5):461–4.
96. Kaoutar Z, Benlemlih A, Taoufiq H, Laila C, Hinde E, Bennis S, et al. Low-grade fibromyxoid sarcoma arising in the big toe. South Med J. 2011;104(3):241–3.
97. Manes RP, Lemeshev Y, Batra PS. Pathology quiz case 2. Low-grade fibromyxoid sarcoma (LGFMS). Arch Otolaryngol Head Neck Surg. 2011;137(2):199, 201–2.
98. Moller E, Hornick JL, Magnusson L, Veerla S, Domanski HA, Mertens F. FUS-CREB3L2/L1-positive sarcomas show a specific gene expression profile with upregulation of CD24 and FOXL1. Clin Cancer Res. 2011;17(9):2646–56.
99. Rekhi B, Deshmukh M, Jambhekar NA. Low-grade fibromyxoid sarcoma: a clinicopathologic study of 18 cases, including histopathologic relationship with sclerosing epithelioid fibrosarcoma in a subset of cases. Ann Diagn Pathol. 2011;15(5):303–11.
100. Lee AF, Yip S, Smith AC, Hayes MM, Nielsen TO, O'Connell JX. Low-grade fibromyxoid sarcoma of the perineum with heterotopic ossification: case report and review of the literature. Hum Pathol. 2011;42(11):1804–9.
101. Laurini JA, Zhang L, Goldblum JR, Montgomery E, Folpe AL. Low-grade fibromyxoid sarcoma of the small intestine: report of 4 cases with molecular cytogenetic confirmation. Am J Surg Pathol. 2011;35(7):1069–73.
102. Kim SK, Jee WH, Lee AW, Chung YG. Haemorrhagic low-grade fibromyxoid sarcoma: MR findings in two young women. Br J Radiol. 2011;84(1003):e146–50.
103. Evans HL. Low-grade fibromyxoid sarcoma: a clinicopathologic study of 33 cases with long-term follow-up. Am J Surg Pathol. 2011;35(10):1450–62.
104. Menon S, Krivanek M, Cohen R. Low-grade fibromyxoid sarcoma, a deceptively benign tumor in a 5-year-old child. Pediatr Surg Int. 2012;28(2):211–3.
105. Rubin G, Rinott M, Wolovelsky A, Elmalach I, Rozen N. Low-grade fibromyxoid sarcoma of the hand: a case report. Hand (N Y). 2010;5(4):449–52.
106. Brasanac D, Dzelatovic NS, Stojanovic M. Giant cystic superficial low-grade fibromyxoid sarcoma. Ann Diagn Pathol. 2013;17(2):222–5.
107. Lam YL, Ho WY, Ng TP, Kan A, Shek TW. A sarcoma of 23 years' duration: symptom duration is not a reliable parameter to exclude malignancy. Hong Kong Med J. 2012;18(3):250–2.
108. Barnhill D, Ismailjee M, Goss N, Ruiz B, Young A. Low-grade fibromyxoid sarcoma of the vulva. J La State Med Soc. 2012;164(2):95–6.
109. Hisaoka M, Matsuyama A, Aoki T, Sakamoto A, Yokoyama K. Low-grade fibromyxoid sarcoma with prominent giant rosettes and heterotopic ossification. Pathol Res Pract. 2012;208(9):557–60.
110. Abe Y, Hashimoto I, Nakanishi H. Recurring facial low-grade fibromyxoid sarcoma in an elderly patient: a case report. J Med Invest. 2012;59(3-4):266–9.
111. Dobin SM, Malone VS, Lopez L, Donner LR. Unusual histologic variant of a low-grade fibromyxoid sarcoma in a 3-year-old boy with complex chromosomal translocations involving 7q34, 10q11.2, and 16p11.2 and rearrangement of the FUS gene. Pediatr Dev Pathol. 2013;16(2):86–90.

112. Odem JL, Oroszi G, Bernreuter K, Grammatopoulou V, Lauer SR, Greenberg DD, et al. Deceptively benign low-grade fibromyxoid sarcoma: array-comparative genomic hybridization decodes the diagnosis. Hum Pathol. 2013;44(1):145–50.
113. Alevizopoulos A, Mygdalis V, Tyritzis S, Stravodimos K, Constantinides CA. Low-grade fibromyxoid sarcoma of the renal pelvis: first report. Case Rep Nephrol Urol. 2012;2(2):87–91.
114. VanSandt AM, Bronson J, Leclair C, Mansoor A, Goetsch M. Low-grade fibromyxoid sarcoma of the vulva: a case report. J Low Genit Tract Dis. 2013;17(1):79–81.
115. Ferlosio A, Doldo E, Polisca P, Orlandi A. Low-grade fibromyxoid sarcoma: an unusual cardiac location. Cardiovasc Pathol. 2013;22(3):e15–7.
116. Dong W, Zhang H. Low-grade fibromyxoid sarcoma of the thyroid: a case report. Ann Acad Med Singap. 2013;42(1):55–6.
117. He KF, Jia J, Zhao YF. Low-grade fibromyxoid sarcoma with cystic appearance and osseous metaplasia in the cheek: a case report and review of the literature. J Oral Maxillofac Surg. 2013;71(6):1143–50.
118. Tominaga Y, Eguchi T, Shiina T, Hamanaka K, Amano J, Asaka S, et al. An intrathoracic low-grade fibromyxoid sarcoma arising from the chest wall with massive pleural effusion. Ann Thorac Cardiovasc Surg. 2014;20 Suppl:509–12.
119. Lau PP, Lui PC, Lau GT, Yau DT, Cheung ET, Chan JK. EWSR1-CREB3L1 gene fusion: a novel alternative molecular aberration of low-grade fibromyxoid sarcoma. Am J Surg Pathol. 2013;37(5):734–8.
120. Bhandarkar V, Kwong K, Micale M, Coticchia J. Pathology quiz case 1: hyalinzed spindle cell tumor with giant rosettes(HSCTGR). JAMA Otolaryngol Head Neck Surg. 2013;139(5):525–7.
121. Furudate S, Fujimura T, Kambayashi Y, Tsukada A, Numata Y, Aiba S. Multiple low-grade fibromyxoid sarcoma on the upper arms with atypical histological presentation. Case Rep Dermatol. 2013;5(2):152–5.
122. Maretty-Nielsen K, Baerentzen S, Keller J, Dyrop HB, Safwat A. Low-Grade Fibromyxoid Sarcoma: Incidence, Treatment Strategy of Metastases, and Clinical Significance of the FUS Gene. Sarcoma. 2013;2013:256280.
123. Singh K, Singh S, Pal N, Sampley SK, Chhabra K. Low-grade fibromyxoid sarcoma of anterior abdominal wall. Indian J Surg. 2012;74(4):351–3.
124. Ferreira CR, da Fonseca LG, Piotto GHM, Geyer FC, de Alcantara PSM. Fibrosarcoma: a challenging diagnosis. Autops Case Rep. 2013;3(3):21–9.
125. Sedrak MP, Parker DC, Gardner JM. Low-grade fibromyxoid sarcoma with nuclear pleomorphism arising in the subcutis of a child. J Cutan Pathol. 2014;41(2):134–8.
126. Alatise OI, Oke OA, Olaofe OO, Omoniyi-Esan GO, Adesunkanmi AR. A huge low-grade fibromyxoid sarcoma of small bowel mesentery simulating hyper immune splenomegaly syndrome: a case report and review of literature. Afr Health Sci. 2013;13(3):736–40.
127. Fritch Lilla SA, Yi JS, Hall BA, Moertel CL. A novel APC gene mutation associated with a severe phenotype in a patient with Turcot syndrome. J Pediatr Hematol Oncol. 2014;36(3):e177–9.
128. Arbajian E, Puls F, Magnusson L, Thway K, Fisher C, Sumathi VP, et al. Recurrent EWSR1-CREB3L1 gene fusions in sclerosing epithelioid fibrosarcoma. Am J Surg Pathol. 2014;38(6):801–8.
129. Thapar S, Ahuja A, Rastogi A. Rare diaphragmatic tumor mimicking liver mass. World J Gastrointest Surg. 2014;6(2):33–7.
130. Kim KJ, Seo JW. Intra-abdominal low-grade fibromyxoid sarcoma of the transverse mesocolon mimicking lymphoma. Jpn J Radiol. 2014;32(6):360–4.
131. Linos K, Bridge JA, Edgar MA. MUC 4-negative FUS-CREB3L2 rearranged low-grade fibromyxoid sarcoma. Histopathology. 2014;65(5):722–4.
132. Alter RY, Wamsley CC, Mullen JT, Haile WZ, Goldsmith JD, Kasper EM. Peripheral nerve fibromyxoid sarcoma. J Neurosurg. 2014;121(3):576–9.
133. Sugita S, Aoyama T, Kondo K, Keira Y, Ogino J, Nakanishi K, et al. Diagnostic utility of NCOA2 fluorescence in situ hybridization and Stat6 immunohistochemistry staining for soft tissue angiofibroma and morphologically similar fibrovascular tumors. Hum Pathol. 2014;45(8):1588–96.

134. Rubinstein JC, Visa A, Zhang L, Antonescu CR, Christison-Lagay ER, Morotti R. Primary low-grade fibromyxoid sarcoma of the kidney in a child with the alternative EWSR1-CREB3L1 gene fusion. Pediatr Dev Pathol. 2014;17(4):321–6.
135. Wojcik JB, Bellizzi AM, Dal Cin P, Bredella MA, Fletcher CD, Hornicek FJ, et al. Primary sclerosing epithelioid fibrosarcoma of bone: analysis of a series. Am J Surg Pathol. 2014;38(11):1538–44.
136. Bajpai J, Shukla S, Jah M, Singh AK, Goel M, Mourya A, et al. Low-grade fibromyxoid sarcoma around the knee involving the proximal end of the tibia and patella: A rare case report. Oncol Lett. 2014;7(4):1308–12.
137. Whale K, Bennett G. Primary pulmonary hyalinising spindle cell tumour with giant rosettes. Pathology. 2014;46(5):451–3.
138. Liang W, Xu S. Imaging findings from a case of pleural low-grade fibromyxoid sarcoma similar to mesothelioma with pleural effusion. Clin Respir J. 2016;10(1):120–4.
139. Citores-Pascual MA, Tinoco-Carrasco C, Arenal-Vera JJ, Benito-Fernandez C, Torres-Nieto Mde L, Zamora-Martinez T. [Low grade fibromixoid sarcoma: a purpose of 3 cases and review of the bibliography]. Cir Cir. 2013;81(4):333–9.
140. Guducu N, Coban I, Bassullu N, Gonenc G, Aydinli K. Low-grade fibromyxoid sarcoma of the vagina: A tumor, not previously reported at this site. Turk J Obstet Gynecol. 2014;11(3):196–7.
141. Kurisaki-Arakawa A, Suehara Y, Arakawa A, Takagi T, Takahashi M, Mitani K, et al. Deeply located low-grade fibromyxoid sarcoma with FUS-CREB3L2 gene fusion in a 5-year-old boy with review of literature. Diagn Pathol. 2014;9:163.
142. Indap S, Dasgupta M, Chakrabarti N, Agarwal A. Low grade fibromyxoid sarcoma (Evans tumour) of the arm. Indian J Plast Surg. 2014;47(2):259–62.
143. Prieto-Granada C, Zhang L, Chen HW, Sung YS, Agaram NP, Jungbluth AA, et al. A genetic dichotomy between pure sclerosing epithelioid fibrosarcoma (SEF) and hybrid SEF/low-grade fibromyxoid sarcoma: a pathologic and molecular study of 18 cases. Genes Chromosomes Cancer. 2015;54(1):28–38.
144. Papp S, Dickson BC, Chetty R. Low-grade fibromyxoid sarcoma mimicking solitary fibrous tumor: a report of two cases. Virchows Arch. 2015;466(2):223–8.
145. Unlu Y, Huq GE, Ozyalvacli G, Zengin M, Koca SB, Yucetas U, et al. Paratesticular sarcomas: A report of seven cases. Oncol Lett. 2015;9(1):308–12.
146. Kurisaki-Arakawa A, Akaike K, Tomomasa R, Arakawa A, Suehara Y, Takagi T, et al. A case of low-grade fibromyxoid sarcoma with unusual central necrosis in a 77-year-old man confirmed by FUS-CREB3L2 gene fusion. Int J Surg Case Rep. 2014;5(12):1123–7.
147. Thway K, Chisholm J, Hayes A, Swansbury J, Fisher C. Pediatric low-grade fibromyxoid sarcoma mimicking ossifying fibromyxoid tumor: adding to the diagnostic spectrum of soft tissue tumors with a bony shell. Hum Pathol. 2015;46(3):461–6.
148. Mendoza AS, O'Leary MP, Peng SK, Petrie BA, Li AI, French SW. Low-grade fibromyxoid sarcoma of the sigmoid colon. Exp Mol Pathol. 2015;98(2):300–3.
149. Chen N, Gong J, Nie L, Chen X, Xu M, Chen M, et al. Primary intracranial low-grade fibromyxoid sarcoma with FUS gene rearrangement. Neuropathology. 2015;35(4):348–53.
150. Varsak YK, Arbag H, Yesildemir HS, Esen H. Low-grade fibromyxoid sarcoma of superior turbinate in a pediatric patient. J Craniofac Surg. 2015;26(3):962–4.
151. Goldstein JA, Cates JM. Differential diagnostic considerations of desmoid-type fibromatosis. Adv Anat Pathol. 2015;22(4):260–6.
152. Thway K, Ng W, Benson C, Chapman J, Fisher C. DOG1 Expression in Low-Grade Fibromyxoid Sarcoma: A Study of 11 Cases, With Molecular Characterization. Int J Surg Pathol. 2015;23(6):454–60.
153. White IK, Scherer AG, Baumanis MM, Abdulkader M, Fulkerson DH. Rapidly enlarging low-grade fibromyxoid sarcoma with intracranial extension in a 5-year-old girl: case report. J Neurosurg Pediatr. 2015;16(4):372–6.
154. Sargar K, Kao SC, Spunt SL, Hawkins DS, Parham DM, Coffin C, et al. MRI and CT of Low-Grade Fibromyxoid Sarcoma in Children: A Report From Children's Oncology Group Study ARST0332. AJR Am J Roentgenol. 2015;205(2):414–20.

155. Konecna J, Liberale G, Haddad J, de Saint-Aubain N, El Nakadi I. Diffuse intra-abdominal low grade fibromyxoid sarcoma with hepatic metastases: Case report and review of the literature. Int J Surg Case Rep. 2015;14:40–3.
156. Cowan ML, Thompson LD, Leon ME, Bishop JA. Low-Grade Fibromyxoid Sarcoma of the Head and Neck: A Clinicopathologic Series and Review of the Literature. Head Neck Pathol. 2016;10(2):161–6.
157. Lee EJ, Hwang HJ, Byeon HK, Park HS, Choi HS. A low grade fibromyxoid sarcoma originating from the masseter muscle: a case report. J Med Case Rep. 2015;9:176.
158. Spalthoff S, Bredt M, Gellrich NC, Jehn P. A Rare Pathology: Low-Grade Fibromyxoid Sarcoma of the Maxilla. J Oral Maxillofac Surg. 2016;74(1):219 e1–10.
159. Benhiba H, Hassam B. [Fibromyxoid sarcoma of unusual location]. Pan Afr Med J. 2015;21:92.
160. Soma S, Bhat S, Shetty SK. Low Grade Fibromyxoid Sarcoma of the Palate: A Case Report. J Clin Diagn Res. 2015;9(10):XD01–XD2.
161. Mazari PM, Weber KL, Kim S, Zhang PJ. Cytogenetically confirmed low-grade fibromyxoid sarcoma arising from the tibia. Hum Pathol. 2016;48:56–9.
162. Lee JH, Choi HJ, Jung HY. Low-Grade Fibromyxoid Sarcoma of the Malar Area. Arch Plast Surg. 2016;43(1):110–2.
163. Zou MX, Lv GH, Wang XB, Li J. Infiltrative low-grade fibromyxoid sarcoma of the thoracic spine. Spine J. 2016;16(9):e573–4.
164. Chien YC, Karolyi K, Kovacs I. Paravertebral Low-grade Fibromyxoid Sarcoma with Supernumerary Ring Chromosome: Case Report and Literature Review. Ann Clin Lab Sci. 2016;46(1):90–6.
165. Shibata S, Shiraishi K, Yamashita H, Kobayashi R, Nakagawa K. Radiation-induced low-grade fibromyxoid sarcoma of the chest wall nine years subsequent to radiotherapy for breast carcinoma: A case report. Oncol Lett. 2016;11(4):2520–4.
166. Chatterjee J, Howden S, Saso S, Ghaem-Maghami S, McIndoe A, Dina R. Metastatic low-grade fibromyxoid sarcoma of the broad ligament: A case report and literature review. J Obstet Gynaecol. 2016;36(7):852–4.
167. Mastoraki A, Strigkos T, Tatakis FP, Christophi A, Smyrniotis V. Recurrent Low-Grade Fibromyxoid Sarcoma of the Neck: Report of a Case and Review of the Literature. Indian J Surg Oncol. 2015;6(3):296–9.
168. Hashimoto M, Koide K, Arita M, Kawaguchi K, Mikuriya Y, Iwata J, et al. A Low-Grade Fibromyxoid Sarcoma of the Internal Abdominal Oblique Muscle. Case Rep Surg. 2016;2016:8524030.
169. Chaudhuri K, Kasimsetty CR, Lingappa A, Gujjar PV. Low-grade fibromyxoid sarcoma involving the mandible: A diagnostic dilemma. J Oral Maxillofac Pathol. 2016;20(2):334.
170. Kesrouani C, Zemoura L, Trassard M, Lae M. [A hybrid lesion: Low-grade fibromyxoid sarcoma (LGFMS) and sclerosing epithelioid fibrosarcoma (SEF)]. Ann Pathol. 2016;36(5):351–4.
171. Sung J, Kim JY. Fatty rind of intramuscular soft-tissue tumors of the extremity: is it different from the split fat sign? Skeletal Radiol. 2017;46(5):665–73.
172. Ban LK, Tseng AH, Huang SH, Lee HH. Low-grade fibromyxoid sarcoma of the external anal sphincter: a case report. World J Surg Oncol. 2017;15(1):109.
173. Mentzel T, Brenn T. Malignant mesenchymal neoplasms of the dermis and subcutis mimicking benign lesions: a case-based review. Virchows Arch. 2017;471(5):565–74.
174. Vallejo-Benitez A, Rodriguez-Zarco E, Carrasco SP, Pereira-Gallardo S, Brugal Molina J, Garcia-Escudero A, et al. Expression of dog1 in low-grade fibromyxoid sarcoma: A study of 19 cases and review of the literature. Ann Diagn Pathol. 2017;30:8–11.
175. Rakheja D, Seaward JR, Timmons CF. Low-Grade Fibromyxoid Sarcoma With Striking Zonation. Int J Surg Pathol. 2018;26(4):332–3.
176. Tan SY, Szymanski LJ, Galliani C, Parham D, Zambrano E. Solitary Fibrous Tumors in Pediatric Patients: A Rare and Potentially Overdiagnosed Neoplasm, Confirmed by STAT6 Immunohistochemistry. Pediatr Dev Pathol. 2018;21(4):389–400.
177. Laliberte C, Leong IT, Holmes H, Monteiro EA, O'Sullivan B, Dickson BC. Sclerosing Epithelioid Fibrosarcoma of the Jaw: Late Recurrence from a Low Grade Fibromyxoid Sarcoma. Head Neck Pathol. 2018;12(4):619–22.

178. Swanson AA, Giannini C, Folpe AL, Van Dyke DL, Amrami KK, Michalak WA, et al. Low-grade fibromyxoid sarcoma arising within the median nerve. Neuropathology. 2018;38(3):309–14.
179. Ud Din N, Ahmad Z, Zreik R, Horvai A, Folpe AL, Fritchie K. Abdominopelvic and Retroperitoneal Low-Grade Fibromyxoid Sarcoma: A Clinicopathologic Study of 13 Cases. Am J Clin Pathol. 2018;149(2):128–34.
180. Wang G, Zhao Z, Wei J, Yang J. Fibromyxoid sarcoma in the retroperitoneum: A case report. Medicine (Baltimore). 2017;96(51):e9409.
181. Mok Y, Pang YH, Sanjeev JS, Kuick CH, Chang KT. Primary Renal Hybrid Low-grade Fibromyxoid Sarcoma-Sclerosing Epithelioid Fibrosarcoma: An Unusual Pediatric Case With EWSR1-CREB3L1 Fusion. Pediatr Dev Pathol. 2018;21(6):574–9.
182. Yue Y, Liu Y, Song L, Chen X, Wang Y, Wang Z. MRI findings of low-grade fibromyxoid sarcoma: a case report and literature review. BMC Musculoskelet Disord. 2018;19(1):65.
183. Zhang Y, Wan D, Gao F. Primary low-grade fibromyxoid sarcoma of the breast: a rare case report with immunohistochemical and fluorescence in situ hybridization detection. Hum Pathol. 2018;79:208–11.
184. Tay TKY, Kuick CH, Lim TH, Chang KTE, Sittampalam KS. A case of low grade fibromyxoid sarcoma with dedifferentiation. Pathology. 2018;50(3):348–51.
185. Park YH, Kim CH, Kim JH, Park JE, Yim SY. Rare Concurrence of Congenital Muscular Torticollis and a Malignant Tumor in the Same Sternocleidomastoid Muscle. Ann Rehabil Med. 2018;42(1):189–94.
186. Geramizadeh B, Zare Z, Dehghanian AR, Bolandparvaz S, Marzban M. Huge mesenteric low-grade fibromyxoid sarcoma: A case report and review of the literature. Rare Tumors. 2018;10:2036361318777031.
187. Bhattar R, Aggarwal SP, Yadav SS, Tomar V. Primary Low-Grade Fibromyxoid Sarcoma of Kidney-an Extremely Rare Entity. Indian J Surg. 2018;80(3):281–3.
188. Alfaro-Cervello C, Benavent Casanova O, Nieto G, Mares Diago FJ, Navarro S. [Low-grade fibromyxoid sarcoma, an essential differential diagnosis in myxoid tumors with benign appearance]. Rev Esp Patol. 2018;51(3):178–82.
189. Han B, Son SJ, Lee JH, Han TY. A Case of Low-Grade Fibromyxoid Sarcoma Arising in the Finger. Ann Dermatol. 2018;30(4):501–2.
190. Karthigeyan M, Malik P, Garg S, Radotra BD, Salunke P, Sunil N, et al. Intracranial Low-Grade Fibromyxoid Sarcoma with Atypical Radiology. World Neurosurg. 2019;122:500–4.
191. Sambri A, Righi A, Tuzzato G, Donati D, Bianchi G. Low-grade fibromyxoid sarcoma of the extremities: a clinicopathologic study of 24 cases and review of the literature. Pol J Pathol. 2018;69(3):219–25.
192. Saab-Chalhoub MW, Al-Rohil RN. Low-grade fibromyxoid sarcoma of acral sites: Case report and literature review. J Cutan Pathol. 2019;46(4):271–6.
193. Seto T, Song MN, Trieu M, Yu J, Sidhu M, Liu CM, et al. Real-World Experiences with Pazopanib in Patients with Advanced Soft Tissue and Bone Sarcoma in Northern California. Med Sci (Basel). 2019;7(3).
194. Rao R, Honavar SG, Mulay K, Reddy VAP. Primary orbital low-grade fibromyxoid sarcoma - A case report. Indian J Ophthalmol. 2019;67(4):568–70.
195. Kumari K, Thota R, Chaudhary HL, Sharma MC, Thakar A, Singh G. Low-Grade Fibromyxoid Sarcoma of the External Auditory Canal: A Rare Pathology and Unusual Location. Head Neck Pathol. 2020;14(1):276–82.
196. Chetverikova E, Kasenomm P. Low-Grade Fibromyxoid Sarcoma of the Lateral Skull Base: Presentation of Two Cases. Case Rep Otolaryngol. 2019;2019:7917040.
197. Scheer M, Vokuhl C, Veit-Friedrich I, Munter M, von Kalle T, Greulich M, et al. Low-grade fibromyxoid sarcoma: A report of the Cooperative Weichteilsarkom Studiengruppe (CWS). Pediatr Blood Cancer. 2020;67(2):e28009.
198. Kanato T, Kalyani S, Lailyang T, Santosh D, Rebecca T, Charai H. Low Grade Fibromyxoid Sarcoma in Oral Cavity: A Rare Case Report. Indian J Otolaryngol Head Neck Surg. 2019;71(Suppl 1):25–6.
199. Fuentes HEM, Monraz-Mendez MF. Low-grade fibromyxoid sarcoma with pulmonary metastasis. A rare case report. Rev Med Inst Mex Seguro Soc. 2020;58(2):221–5.
200. Shimizu Y, Tsuchiya K, Fujisawa H. Intracranial Low-Grade Fibromyxoid Sarcoma: Findings on Electron Microscopy and Histologic Analysis. World Neurosurg. 2020;135:301–5.

201. Puls F, Agaimy A, Flucke U, Mentzel T, Sumathi VP, Ploegmakers M, et al. Recurrent Fusions Between YAP1 and KMT2A in Morphologically Distinct Neoplasms Within the Spectrum of Low-grade Fibromyxoid Sarcoma and Sclerosing Epithelioid Fibrosarcoma. Am J Surg Pathol. 2020;44(5):594–606.
202. Zhao MM, Li CS, Jun W, Cheng YP, Zhao DX. A case report of paravertebral low-grade malignant fibrous myxoid sarcoma. Medicine (Baltimore). 2020;99(3):e18800.
203. Kramer SP, Bowman CJ, Wang ZJ, Sheahon KM, Nakakura EK, Cho SJ, et al. Hybrid Low-Grade Fibromyxoid Sarcoma and Sclerosing Epithelioid Fibrosarcoma of the Pancreas. J Gastrointest Cancer. 2020;51(3):1025–9.
204. Huang J, Cohen S, Jour G. Primary small intestine mesenteric low-grade fibromyxoid sarcoma with foci of atypical epithelioid whorls and diffuse DOG1 expression: a case report. Diagn Pathol. 2020;15(1):23.
205. Mustafa S, VandenBussche CJ, Ali SZ, Siddiqui MT, Wakely PE, Jr. Cytomorphologic findings of low-grade fibromyxoid sarcoma. J Am Soc Cytopathol. 2020;9(3):191–201.
206. Logan SJ, Perricone A, Farris AB, Edgar M. Low-grade fibromyxoid sarcoma: a potentially useful histologic finding. Histopathology. 2020;77(2):329–31.
207. Murshed KA, Ammar A. Hybrid sclerosing epithelioid fibrosarcoma/low grade fibromyxoid sarcoma arising in the small intestine with distinct HEY1-NCOA2 gene fusion. Pathology. 2020;52(5):607–10.
208. Koucky V, Kalfert D, Kodetova Novakova D, Plzak J. Low-grade fibromyxoid sarcoma of the maxillary sinus. Biomed Pap Med Fac Univ Palacky Olomouc Czech Repub. 2021;165(3):342–5.
209. Perez D, El-Zammar O, Cobanov B, Naous R. Low-grade fibromyxoid sarcoma: A rare case in an unusual location. SAGE Open Med Case Rep. 2020;8:2050313X20944315.
210. Yuan M, Wang R, Zhang F, Wang L. Low-grade fibromyxoid sarcoma in inguinal region: A case report. Asian J Surg. 2020;43(12):1189–90.
211. Chitayat S, Barros R, Ribeiro JG, Silva HAM, Sa FR, Reis BSB, et al. Case Report: An extremely rare occurrence of recurrent inguinal low-grade fibromyxoid sarcoma involving the scrotum. F1000Res. 2020;9:789.
212. Kosemehmetoglu K, Ardic F, Kilpatrick SE, Aydingoz U, Sumathi VP, Michal M. Sclerosing epithelioid fibrosarcoma of bone: morphological, immunophenotypical, and molecular findings of 9 cases. Virchows Arch. 2021;478(4):767–77.
213. Righi A, Pacheco M, Pipola V, Gambarotti M, Benini S, Sbaraglia M, et al. Primary sclerosing epithelioid fibrosarcoma of the spine: a single-institution experience. Histopathology. 2021;78(7):976–86.
214. Dugalic V, Ignjatovic, II, Kovac JD, Ilic N, Sopta J, Ostojic SR, et al. Low-grade fibromyxoid sarcoma of the liver: A case report. World J Clin Cases. 2021;9(1):175–82.
215. Oramas DM, Alqaidy D, Moran CA. Primary pulmonary hyalinizing spindle cell tumor with giant rosettes: A clinicopathological and immunohistochemical study of 2 cases. Ann Diagn Pathol. 2021;51:151706.
216. Sajid MI, Arshad S, Abdul-Ghafar J, Fatimi SH, Din NU. Low-grade fibromyxoid sarcoma incidentally discovered as an asymptomatic mediastinal mass: a case report and review of the literature. J Med Case Rep. 2021;15(1):50.
217. Auria-Aspiazu D, Parrales-Calderon O, Moncayo-Asnalema F. Low-grade fibromyxoid sarcoma as a content of perineal hernia. A case report. Cir Cir. 2021;89(2):248–51.
218. Kiyohara T, Tanimura H. Intramuscular Low-Grade Fibromyxoid Sarcoma: An Efficacy of Cytoplasmic Mucin 4 Immunoexpression. Ann Dermatol. 2020;32(4):350–2.
219. Deewani MH, Danish MH, Awan MS, Ud Din N. Low-grade fibromyxoid sarcoma of the parapharyngeal space: an unusual location. BMJ Case Rep. 2021;14(5).
220. Niepokoj-Czopnik A, Aporowicz M, Halon L, Maciejczyk A, Matkowski R. Low-grade fibromyxoid sarcoma of a male breast: a uniquely rare case report. Pol Arch Intern Med. 2021;131(7-8):724–6.
221. Chivchibashi DL, Pavlov P, Tzaneva M, Sapundzhiev N, Davidov G. Radiation-induced low grade fibromyxoid sarcoma of the larynx: a case report and literature review. Folia Med (Plovdiv). 2021;63(3):433–7.
222. Sharma A, Thangaiah JJ, Shetty S, Policarpio-Nicolas MLC. Bone and soft tissue sarcomas in cerebrospinal fluid and effusion: A 20-year review at our institution. Cancer Cytopathol. 2021;129(10):776–87.
223. Williams CM, Du W, Mangano WE, Mei L. Mediastinal Low-Grade Fibromyxoid Sarcoma With FUS-CREB3L2 Gene Fusion. Cureus. 2021;13(6):e15606.

224. Yoshimura R, Nishiya M, Yanagawa N, Deguchi H, Tomoyasu M, Kudo S, et al. Low-grade fibromyxoid sarcoma arising from the lung: A case report. Thorac Cancer. 2021;12(18):2517–20.
225. Naik VG, Rai KK, Shivakumar HR. Low-grade fibromyxoid sarcoma: A rare case report. Natl J Maxillofac Surg. 2021;12(2):271–5.
226. Yoshimatsu Y, Noguchi R, Sin Y, Tsuchiya R, Ono T, Sei A, et al. Establishment and characterization of NCC-LGFMS1-C1: a novel patient-derived cell line of low-grade fibromyxoid sarcoma. Hum Cell. 2021;34(6):1919–28.
227. Gjorgova Gjeorgjievski S, Fritchie K, Thangaiah JJ, Folpe AL, Din NU. Head and Neck Low-Grade Fibromyxoid Sarcoma: A Clinicopathologic Study of 15 Cases. Head Neck Pathol. 2021.
228. Cantu NA, Ullah A, Stumpo-Decoons L, Belakhlef S, Kruse EJ. Low-Grade Fibromyxoid Sarcoma of the Back. Cureus. 2021;13(8):e17308.
229. Tian K, Johnstone K, Lambie D, Frankel A. Low-grade fibromyxoid sarcoma with high-grade features, a rare finding. ANZ J Surg. 2021.
230. Patton A, Bridge JA, Liebner D, Chung C, Iwenofu OH. A YAP1::TFE3 cutaneous low-grade fibromyxoid neoplasm: A novel entity! Genes Chromosomes Cancer. 2021.
231. Zhang M, Yu Y, Guan X, Yao X, Jia C, Hong E, et al. A group of sclerosing epithelioid fibrosarcomas with low-level amplified EWSR1-CREB3L1 fusion gene in children. Pathol Res Pract. 2022;230:153754.
232. Froiio C, Berlth F, Capovilla G, Tagkalos E, Hadzijusufovic E, Mann C, et al. Robotic-assisted surgery for esophageal submucosal tumors: a single-center case series. Updates Surg. 2022.
233. Chan YC, Kan ANC, Yuen LYP, Wan IYP, Fung KKF, Cheung YF, et al. Case Report: Primary Thoracic Low-Grade Fibromyxoid Sarcoma in a Young Girl Presenting With Mediastinal Mass Syndrome. Front Pediatr. 2022;10:885068.
234. Nunes Pombo J, Nixon Martins A, Paias Gouveia C, Nawojowska Á, Mendes S, Cabral D, et al. Rare case of low-grade fibromyxoid sarcoma of the thoracic wall with complete sternum reconstruction. Case Reports Plast Surg Hand Surg. 2022;9(1):126–30.
235. Khamashta N, Dalal A, Alashwas M, Idkedek M, Abu-Akar F. Case report and review of literature: Resection of a huge mediastinal low-grade fibromyxoid sarcoma with neck, axillary, and lung involvement. Front Surg. 2022;9:988881.
236. Costigan D, Dal Cin P, Fletcher CDM, Nucci MR, Parra-Herran C, Chapel DB. Low-grade Fibromyxoid Sarcoma of the Vulva and Vagina: Clinical, Pathologic, and Molecular Characterization of 7 Cases and Review of the Literature. Am J Surg Pathol. 2022;46(9):1196–206.
237. Guo Y, Hao Y, Guan G. Low-grade fibromyxoid sarcoma in the middle ear as a rare location: a case report. Transl Pediatr. 2022;11(6):1034–9.
238. Xie Y, Wang S, Yan D, Shen J. Primary low-grade fibromyxoid sarcoma of the mediastinum: A case report. Asian J Surg. 2022;45(10):2150–1.
239. Ershadi R, Vahedi M, Jahanbin B, Tabatabaei FS, Rafieian S. Giant primary low-grade fibromyxoid sarcoma arising from the left pulmonary parenchyma: A case report and literature review. Cancer Rep (Hoboken). 2022;5(11):e1718.
240. Sahraoui G, Sassi F, Charfi L, Ghallab M, Mrad K, Doghri R. Low-grade fibromyxoid sarcoma of the vulva presenting as a cystic mass: A case report and review of literature. Int J Surg Case Rep. 2022;100:107736.
241. Tan S, Liu H, Pan E, Liu S, Zhang J, Wang J, et al. Comprehensive next-generation sequencing reveals low-grade fibromyxoid sarcoma of the vulva missed by morphological diagnosis: a case report. Front Med (Lausanne). 2023;10:1343407.
242. Ronen S, Ko JS, Rubin BP, Kilpatrick SE, Wang WL, Lazar AJ, et al. Superficial low-grade fibromyxoid sarcoma. J Cutan Pathol. 2023;50(2):147–54.
243. He X, Jing W, He X, Chen M, Zhang H. Case report: Primary pleural low-grade fibromyxoid sarcoma in a 4-year-old boy with molecular confirmation. Front Oncol. 2023;13:1269078.
244. Muñoz-Leija MA, Alemán-Jiménez MC, Plata-Álvarez H, Menes-Ramírez G. Low-Grade Fibromyxoid Sarcoma of the Abdominal Wall: A Clinical Case Report. Cureus. 2023;15(3):e35699.

245. Lavin L, Krishnamurthy N, Grant CS, Bailey K. A unique case of hybrid sclerosing epithelioid fibrosarcoma low-grade fibromyxoid sarcoma with EWSR1-CREB3L1 fusion in an 11 year-old treated with robotic-assisted bronchoscopy. Pediatr Blood Cancer. 2023:e30477.
246. Weiler EJ, Murickan T, Drayer CN, Samat SH, Kia MA. A rare case report of pancreatic low-grade fibromyxoid sarcoma (LGFMS). Surg Case Rep. 2023;9(1):123.
247. Narukami E, Anayama T, Yamamoto M, Bunno Y, Miyazaki R, Okada H, et al. Rapidly developing intrathoracic low-grade fibromyxoid sarcoma: A case report. Thorac Cancer. 2023;14(23):2314–9.
248. Otieno DO, Malik J, Wabwire B. Pediatric patient with a complex Abdominal Wall defect reconstructed with sandwich omental flap. A case report. Int J Surg Case Rep. 2023;109:108512.
249. Theodorou DJ, Theodorou SJ, Kakitsubata Y. Fibrosarcoma with deceptive benign presentations: a report of two cases. Folia Med (Plovdiv). 2023;65(4):686–92.
250. Cloutier JM, Moreland A, Wang L, Kunder CA, Allard G, Wang A, et al. Low-grade fibromyxoid sarcoma of the breast: genetic characterization and immunohistochemical comparison to morphologic mimics. Hum Pathol. 2023;139:17–26.
251. Basiri A, Montazeri P, Dadpour M. Penoscrotal Low-grade fibromyxoid sarcoma, A case report. Urol Case Rep. 2023;50:102499.
252. Nawojowska Á, Mendes S, Cabral D, Antunes M, Félix F. Innovative 3d-Printed Prosthesis In A Rare Case Of A Huge Mass Of Anterior Thoracic Wall - Low Grade Fibromyxoid Sarcoma (Lgfms). Port J Card Thorac Vasc Surg. 2023;30(3):81–4.
253. Tasaki T, Shiba E, Noguchi H, Kirishima M, Kitazono I, Terabaru W, et al. Low-grade Fibromyxoid Sarcoma With Massive Degeneration: A Case of Unusual Gross and Histological Features. In Vivo. 2023;37(6):2863–8.
254. Anehosur V, Kumar N, Visweswaran A, Kumar K, Prabhu A. Low-Grade Fibromyxoid Sarcoma in the Hard Palate: A Rare Case Report and Review of the Literature. J Maxillofac Oral Surg. 2023;22(4):1180–5.
255. Johnson M, Silberstein H, Korones D, Hussain A, Hawes D. Low-grade fibromyxoid tumor of the dura: A new entity? Surg Neurol Int. 2024;15:14.
256. Ayadi A, Houcine Y, Moussa C, Rouis H, Abid T, Hugues B, et al. Primary lung low-grade fibromyxoid sarcoma: A rare case with A diagnostic dilemma. Rare Tumors. 2024;16:20363613241234201.
257. Zhang X, Qiu Y, Zhang J, Chen Z, Yang Q, Huang W, et al. An elderly low-grade fibromyxoid sarcoma patient with early postoperative recurrences and metastases: a case report and literature review. Front Med (Lausanne). 2024;11:1172746.
258. Watanabe H, Nakanishi K, Ueno H, Kato T, Shimoyama Y, Chen-Yoshikawa TF. Thoracoscopic Wedge Resection for Low-Grade Fibromyxoid Sarcoma (Evans Tumor) with Massive Calcification and Originating from the Lung: A Rare Case in an Unexpected Location. Ann Thorac Cardiovasc Surg. 2024;30(1).
259. Ishida H, Funaki S, Taniguchi S, Morii E, Shintani Y. Familial multiple endocrine neoplasia type 1 with intrathoracic low-grade fibromyxoid sarcoma. Surg Case Rep. 2024;10(1):16.
260. Suster DI, Gross JM, Fayad L, Wenokor C, Goldsmith JD, Ward A, et al. Sclerosing epithelioid fibrosarcoma of bone with hybrid features: clinicopathologic, radiologic, and molecular analysis of three cases. Skeletal Radiol. 2024;53(2):387–93.
261. Alkameshki M, Nail LL, Tallegas M, Miquelestorena-Standley E, Samargandi R. A Rare Coexistence of Hybrid Tumor Low-grade Fibromyxoid Sarcoma/Sclerosing Epithelioid Fibrosarcoma and Hibernoma in the Same Thigh: A Case Report. J Orthop Case Rep. 2024;14(3):130–5.
262. Tally H, Al-Janabi MAH, AlDwairy H, Al-Shehabi Z, Ibrahim M. Low-grade fibromyxoid sarcoma in laryngopharynx: the first case report in the literature. J Surg Case Rep. 2024;2024(3):rjae141.
263. Barger A, Vishnia M, Hanna M, Horgan R, Martins J. Abdominal Fibromyxoid Sarcoma in Pregnancy: An Unusual Cause of Preterm Labor and Sepsis. AJP Rep. 2024;14(2):e188–e92.
264. Noguchi T, Suzuki T, Shibata Y, Koizumi M, Osaka K, Kishida T. Retroperitoneal low-grade fibromyxoid sarcoma. IJU Case Rep. 2024;7(3):255–8.
265. Quiceno E, Soliman MAR, Khan A, Cavagnaro MJ, McSpadden RP, Pollina J, et al. Supraclavicular Artery Island Flap for Treatment of Cervical Wound Defects and Persistent Cerebrospinal Fluid Leaks: A Technical Note and Systematic Review of the Literature. World Neurosurg. 2024;185:e915–e25.

266. Oh AJ, Singh P, Pirakitikulr N, Roelofs K, Glasgow BJ, Rootman DB. Low-grade fibromyxoid sarcoma of the orbit. Orbit. 2024;43(3):375–9.
267. Fernández J, Alconchel F, Frutos MD, Gil E, Gómez-Valles P, Gómez B, et al. Combined use of composite mesh and acellular dermal matrix graft for abdominal wall repair following tumour resection. World J Surg Oncol. 2024;22(1):226.
268. Suleman M, Lodhia J, Mremi A, Wampembe E, Uisso F, Chilonga K. A rare case of low-grade fibromyxoid sarcoma of the thigh: Diagnostic and therapeutic challenges in a resource-limited setting. Int J Surg Case Rep. 2025;129:111218.
269. Jia X, Ma R, Liu H, Zhang B. Low-Grade Fibromyxoid Sarcoma Incidentally Discovered as a Hip Mass: A Case Report. J Clin Ultrasound. 2025;53(5):1160–5.
270. Salinas-Castro KJ, Mejía-Quiñones V, García CIC, Zuñiga-Londoño NY. Low-grade fibromyxoid sarcoma of the liver in a pediatric patient: Case report. Radiol Case Rep. 2025;20(9):4778–84.
271. Kubetsky YE, Labzina EE, Kosimshoev MA, Kholtobin DP, Khalepa RV, Nabiev AO, et al. Laparoscopic resection of neurogenic presacral tumors. Zh Vopr Neirokhir Im N N Burdenko. 2025;89(1):59–67.
272. Kinoshita T, Kito M, Kato H, Miyaoka S, Okamoto M, Iwaya M. Microsurgical Reconstruction After Unplanned Resection of the Low-grade Fibromyxoid Sarcoma at the Thumb Base: A Case Report. JBJS Case Connect. 2025;15(3).
273. Mohamedali R, Nishith N, Raj R, Sharma A, Somal PK, Pawar RN, et al. Low-grade fibromyxoid sarcoma, a rare tumor at an unusual site: Case report and review of literature. Discoveries (Craiova). 2025;13(1):e209.
